# LdtR is a master regulator of gene expression in *Liberibacter asiaticus*

**DOI:** 10.1111/1751-7915.12728

**Published:** 2017-05-15

**Authors:** Fernando A. Pagliai, Janelle F. Coyle, Sharan Kapoor, Claudio F. Gonzalez, Graciela L. Lorca

**Affiliations:** ^1^ Department of Microbiology and Cell ScienceGenetics InstituteInstitute of Food and Agricultural SciencesUniversity of Florida2033 Mowry RoadPO Box 103610GainesvilleFL32610‐3610USA

## Abstract

Huanglongbing or citrus greening disease is causing devastation to the citrus industry. *Liberibacter asiaticus*, an obligate intracellular pathogen of citrus, is one the causative agents of the disease. Most of the knowledge about this bacterium has been deduced from the *in silico* exploration of its genomic sequence. *L. asiaticus* differentially expresses genes during its transmission from the psyllid vector, *Diaphorina citri*, to the plant. However, the regulatory mechanisms for the adaptation of the bacterium into either hosts remain unknown. Here we show that LdtR, a MarR family transcriptional regulator, activates or represses transcription genome‐wide. We performed a double approach to identify the components of the LdtR regulon: a transcriptome analysis in both the related bacterium *Liberibacter crescens* and citrus‐infected leaves, strengthened with an *in silico* prediction of LdtR regulatory sites. Our results demonstrated that LdtR controls the expression of nearly 180 genes in *L. asiaticus*, distributed in processes such as cell motility, cell wall biogenesis, energy production, and transcription. These results provide new evidence about the regulatory network of *L. asiaticus*, where the differential expression of genes from these functional categories could be of great importance during the adaptation of the bacterium to either hosts.

## Introduction

For nearly a decade, the phloem‐limited α‐proteobacteria *Liberibacter asiaticus* has caused devastation to Florida's citrus groves (Hodges and Spreen, 2015). The development of more effective antimicrobials against this pathogen has been hampered in part by the inability to culture this microorganism in a laboratory setting. As with many intracellular pathogens, *L. asiaticus* has undergone reductive evolution (Konstantinidis and Tiedje, 2004; Hartung *et al*., 2011). *In silico* analysis revealed that *L. asiaticus* encodes a large number of genes involved in cell motility and active transport of molecules, whereas it encodes a small number of genes involved in biosynthetic pathways or regulatory elements (Duan *et al*., 2009). It also encodes a large number of genes from uncharacterized pathways and hypothetical genes. This bacterium does not encode genes for type II, III or IV secretion systems, commonly associated as determinants of virulence (Duan *et al*., 2009). Therefore, a better understanding of the regulatory pathways that leads the bacterium to survive in its different hosts is an auspicious approach to develop new strategies against citrus greening disease. Comparative genomic analyses indicated that *L. asiaticus* possesses a simple regulatory network, with only 11 transcription factors controlling the expression of the entire transcriptome (Duan *et al*., 2009). Six of these genes encoding for transcription factors were up‐regulated when the bacteria are located in the plant compared to the psyllid host (Yan *et al*., 2013). Among this group of up‐regulated transcriptional regulators *in planta* is LdtR, the only member of the MarR family present in the genome of *L. asiaticus*. This family comprises a diverse group of transcription factors traditionally involved in multidrug resistance (Wilkinson and Grove, 2006). Although more than 12,000 MarR proteins have been identified in bacterial and archaeal genomes, the physiological role of only a small percentage has been elucidated (Kumarevel, 2012). Some of these characterized MarR family members have been associated as sensors of environmental changes within a variety of physiological roles, such as sensing of sugars, redox balance, virulence and degradation of toxic compounds as well as multidrug resistance (Cohen *et al*., 1993; Reverchon *et al*., 1994; Ludwig *et al*., 1995; Sulavik *et al*., 1995; Stapleton *et al*., 2002; Bartels *et al*., 2003; Panmanee *et al*., 2006; Perera and Grove, 2010).

Recently, a novel role in the mediation of osmotic stress tolerance as well as the regulation of a cell wall modification enzyme has been proposed for LdtR (Pagliai *et al*., 2014). Using a small molecule screening assay (Vedadi *et al*., 2006), we were able to identify high‐affinity ligands for LdtR, such as benzbromarone and phloretin. Benzbromarone is an uricosuric drug in mammals and non‐competitive analogue of the xanthine oxidase (Heel *et al*., 1977); whereas phloretin is a dihydrochalcone flavonoid widely distributed in apple trees (Barreca *et al*., 2014) that has been proposed as a enhancer for skin‐based drug delivery (Auner *et al*., 2003). The interaction between these small molecules and LdtR resulted in an inhibition of the binding of LdtR to its cognate promoters as well as a decreased transcriptional activity of *L. asiaticus* in an infected citrus shoots model (Pagliai *et al*., 2014).

The pathways involved in the colonization of L*. asiaticus* in either the citrus or the psyllid host remain unknown. As gene expression in *L. asiaticus* is controlled by just a few transcription factors, we sought to determine whether LdtR is involved in the regulation of gene expression genome‐wide. Therefore, the goal of this study was to elucidate and identify the members of the LdtR regulon in *L. asiaticus*, through a chemical modulation of the LdtR activity by small molecules. Due to the lack of efficient genetic systems, the use and delivery of high‐affinity ligands as a mechanism of protein inactivation offer an alternative approach to increase the understanding of *L. asiaticus*’ physiology, with the overall objective of developing new therapeutic methods against citrus greening disease.

## Results

### LdtR ligands modify *Liberibacter crescens* BT‐1 gene expression profiling

**Figure 1:**
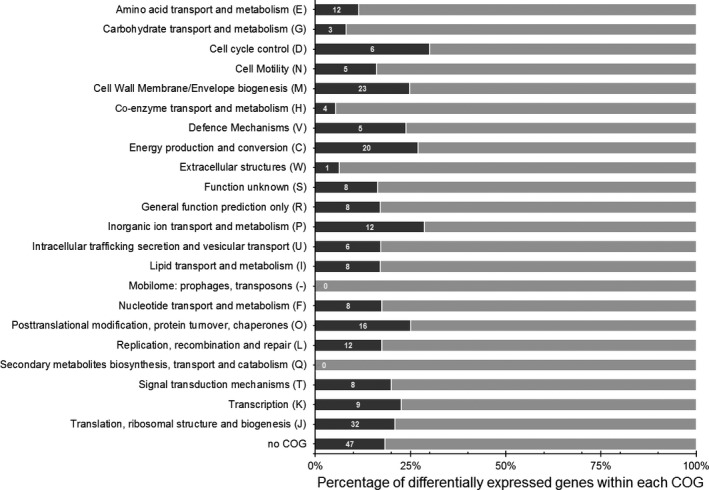
Functional classification of the differentially expressed genes in cells of *L. crescens* treated with phloretin based on the Cluster of Orthologues Genes (COG). The percentage of differentially expressed genes within each COG was calculated as the number of hits from a given category in the RNA‐seq experiments divided by the total number of genes present within that COG in the genome of *L. crescens* BT‐1. The number of hits from each COG category is also indicated.

A subset of mRNA sequencing assays to identify LdtR‐regulated genes was performed. The phylogenetically related bacterium *Liberibacter crescens* was used as a surrogate model strain due to the impossibility of maintaining axenic *L. asiaticus* cultures in laboratory settings. To identify the members of the LdtR regulon, we introduce a shift in the gene expression by culturing the cells in the presence of well‐known LdtR ligands (Pagliai *et al*., 2014). *L. crescens* cells were grown to exponential phase in the presence or absence of sublethal concentrations of phloretin (20 μM) or benzbromarone (50 μM) (Pagliai *et al*., 2014). After sequencing the mRNA transcripts, we identified a group of 252 differentially expressed genes in cells treated with phloretin (*p*adj value ≤ 0.05). In the cells cultured with benzbromarone, only 86 genes were differentially expressed (*p*adj value ≤ 0.05). Remarkably, all of the differentially expressed genes in *L. crescens* treated with benzbromarone were also affected in the phloretin treatment. A strong correlation was found between the changes in mRNA abundance in cells grown in the presence of benzbromarone or phloretin, as determined by the Pearson's coefficient (0.96) and the R‐squared (0.92; Fig. S1). As both ligands are known to trigger a similar LdtR response (Pagliai *et al*., 2014), we hypothesized that this difference could be consequence of a better uptake of phloretin. Therefore, to maximize the number of recovered genes, we further analysed the transcriptome changes triggered by the phloretin treatment. All the genes differentially expressed due to the addition of phloretin were identified using Blast2Go (Conesa *et al*., 2005). To improve the reading and analysis of the obtained data, the information was organized in groups following the Cluster of Orthologues Genes (COGs) categories. These results are summarized in Table S1. Most of the differentially expressed genes showed 1.3‐fold change to twofold change (induction or repression). This relatively low change in gene expression was expected as we used sublethal concentrations of the small molecules (Pagliai *et al*., 2014). Another possible contributing factor to these values could be the high duplication time for *L. crescens* (approximately 24 h), which may result in low transcriptional activity. Among these differentially expressed genes, 131 were up‐regulated, whereas 121 were down‐regulated (Table S1). UDP‐3‐O‐acyl‐*N*‐acetylglucosamine deacetylase (*lpxC*,* B488_05980*), the zinc ABC transporter (*znuA*,* B488_07960*), and the β‐(1‐>2)glucan export ATP‐binding/permease protein (*ndvA*,* B488_01280*) were the highest induced genes in our experimental conditions. Instead, the hypothetical protein *B488_RS05770*, the translation elongation factor TU (*B488_04080*), an uncharacterized ABC transporter (*B488_12120*), together with a LuxR family transcriptional regulator (*B488_01800*) were the most down‐regulated genes due to the chemical inactivation of LdtR. A further analysis showed that the differentially expressed genes (up‐ and down‐regulated) were involved in a myriad of cellular processes (Fig. 1), such as cell cycle control (Group D, 30%); inorganic ion transport and metabolism (Group P, 29%); energy production and conversion (Group C, 27%); cell wall membrane biogenesis (Group M, 25%); post‐translational modification and chaperones (Group O, 25%); defence mechanisms (Group V, 24%); and transcription (Group K, 23%). This scattered regulatory pattern is consistent with the large phenotypic changes observed in *Sinorhizobium meliloti* Δ*ldtR* strain or upon chemical inactivation of LdtR in *L. crescens* (Pagliai *et al*., 2014). This assertion was reinforced after analysing the RNA‐seq data with the BOG algorithm (Park *et al*., 2015), which analyses the number of hits in function of the *p*adj value obtained from the DESeq2 analysis. Interestingly, it was found that genes involved in defence mechanisms (Group V), cell wall membrane biogenesis (Group M) and transcription (Group K) were the preferential targets of LdtR, and they were enriched as a result of the phloretin treatment.

**Figure 2:**
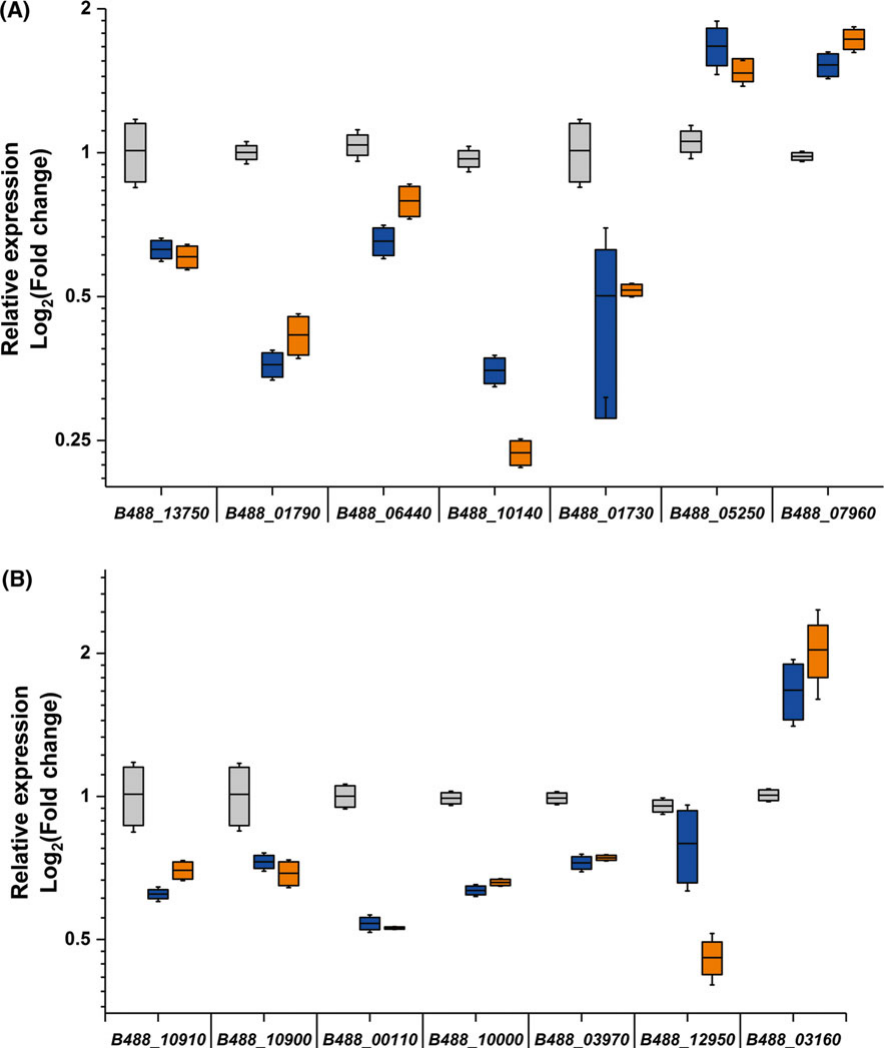
The chemical inactivation of LdtR modifies the expression profile of several functional categories in *L. crescens*. The expression levels of a subset of genes identified in (A) RNA‐seq experiments, or (B) RegPredict, were assessed to monitor the transcriptional activity of LdtR upon inactivation by benzbromarone (blue boxes) or phloretin (orange boxes). Grey boxes represent control conditions. The horizontal line in each box plot represents the mean value. The relative expression values of each gene were obtained by normalizing their levels against the expression levels of 16S rRNA of *L. crescens*. The changes in expression for every gene due to the addition of benzbromarone or phloretin displayed statistical significance (*P *<* *0.05) compared to control conditions according to ANOVA. The experiments were performed in quadruplicates.

The results observed in the transcriptome analysis were confirmed by measuring the expression of a subset of genes by qRT‐PCR. The selected genes for this new analysis were *B488_13750*, encoding for the septum formation inhibitor Maf; *B488_01790*, for a LuxR family transcriptional regulator; *B488_06440*, a kinesin‐like protein; *B488_10140* and *B488_07960*, representing the metal ABC transporters; *B488_01730*, for a ferredoxin; and *B488_05250*, encoding for a glutamate racemase. As expected, all these selected genes followed the same regulatory pattern observed during the RNA‐seq assays (up‐regulated or down‐regulated due to phloretin or benzbromarone treatment, Fig. 2A). The transcript levels of the glutamate racemase and the zinc ABC transporter (*B488_07960*) were increased by 160% and 175% respectively, while a decrease in the transcript levels of the septum formation inhibitor Maf, the LuxR family transcriptional regulator, the kinesin‐like protein, the metal ABC transporter (*B488_10140*) and the ferredoxin was observed. The ability to repress gene expression, triggered by the selected ligands, represents a new characteristic for LdtR. Altogether, these results suggest that this global regulator preferentially down‐regulates genes associated to groups V, C and O, whereas it up‐regulates genes from groups N and S (Table S1).

### Identification of the LdtR regulon in *L. asiaticus*

**Figure 3:**
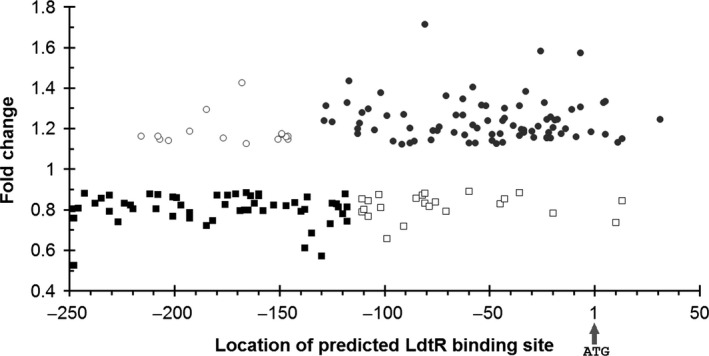
Location of the LdtR binding sequence in the promoter region of *L. asiaticus* genes. The location of the LdtR binding site was predicted for every homologue in *L. asiaticus* of the differentially expressed genes in *L. crescens* using RegPredict (Pagliai *et al*., 2014). The genes were clustered using K‐means (Hartigan and Wong, 1979). The fold change of the transcript levels of the RNA‐seq experiments in *L. crescens* and the location of the predicted LdtR binding site in *L. asiaticus* are shown. In grey circles, genes up‐regulated under phloretin treatment with a centre at the position −52. In black squares, genes down‐regulated under phloretin treatment with a centre at the position −190. Empty symbols represent the genes whose predicted binding site was not clustered between the two main groups. The location of the binding site is relative to the translational start site (indicated with an arrow at the position 1).

Although *L. crescens* is one of the closest culturable phylogenetic bacterium, the results obtained using this surrogate should not be directly extrapolated to elucidate regulatory aspects in *L. asiaticus*. The genome of *L. crescens* shares 832 genes with *L. asiaticus,* which still encodes 532 unique genes (Fagen *et al*., 2014). Of the 252 differentially expressed genes identified in the *L. crescens* RNA‐seq experiments, 182 were found to have a putative homologue in *L. asiaticus*. In parallel to the RNA‐seq assay, we performed a genome‐wide *in silico* analysis in *L. asiaticus* to identify potential LdtR binding sequences. This search was accomplished using the computational platform RegPredict (Novichkov *et al*., 2010). The software was directed to identify sequences with high similarity to the LdtR binding motif (5’ ATATTCCTTGTATTTTAA 3’) determined earlier by DNase I footprinting (Pagliai *et al*., 2014) *. *A stringent cut‐off was used to maximize the accuracy of the predictions, and the area to search was defined in a range of 300 nucleotides, from positions −250 to +50, using the translational start codon as a reference. RegPredict identified 235 genes encoding an LdtR binding sequence following those parameters, 182 of which overlapped with the genes retrieved by the RNA‐seq analysis. Interestingly, it was found that the LdtR binding sites were located at different positions throughout all of the evaluated promoters. Subsequently, we use the R‐package K‐means (Hartigan and Wong, 1979) to cluster these genes based on two criteria: (i) the fold change of the transcript levels during the RNA‐seq experiments and (ii) the location of the predicted LdtR binding site in the homologues from *L. asiaticus*. Four clear clusters were identified (Fig. 3); the majority of the genes up‐regulated due to the phloretin treatment (81 of 96 genes) were clustered with a centre at the −52 position with respect to the start codon. This is consistent with the canonical location of binding motifs frequently used by MarR family members repressing gene expression (Martin and Rosner, 1995). On the other hand, the majority of the genes down‐regulated once LdtR was inactivated by phloretin (62 of 86 genes) were clustered with a centre at the −190 position. This suggests that those genes require LdtR to be transcribed, and the transcriptional regulation of this group of genes is in agreement with the regulatory mechanism originally described for the *ldtP* and *ldtR* promoters (Pagliai *et al*., 2014).

The *in silico* approach identified 53 extra genes encoding a putative LdtR binding site over the selected region (Table S2). Either we did not detect significant changes in expression in homologues of these genes in the RNA‐seq experiments in *L. crescens*, or the surrogate does not have a homologue encoded in its genome. To confirm if LdtR regulates those genes, a subset of the genes was selected to measure their expression levels via qRT‐PCR in *L. crescens*. The selected genes were *B488_10910*, encoding for LdtR_Lcr_; *B488_10900*, for LdtP_Lcr_; *B488_00110*, the DNA translocase FtsK; *B488_10000*, a dTDP‐4‐dehydrorhamnose 3,5‐epimerase; *B488_03970*, a metallophosphatase; *B488_12950*, encoding for LotP; and *B488_03160*, encoding for an alanine racemase. Others genes encoding putative or hypothetical proteins were not selected; however, they are listed in Table S2. It was found that the mRNA levels of *ldrR*_*Lcr*_, *ldtP*_*Lcr*_, the DNA translocase FtsK, the dTDP‐4‐dehydrorhamnose 3,5‐epimerase, the metallophosphatase and LotP were significantly (*P *<* *0.05) decreased (40%, 30%, 50%, 40%, 30% and 60% respectively); whereas the mRNA level of the alanine racemase was significantly (*P *<* *0.05) increased (160%) due to the presence of LdtR ligands (Fig. 2B). These results suggest the transcripts of these genes could be of low abundance in the samples and difficult to select as differentially regulated in the whole transcriptome analysis. In summary, these results suggest that LdtR acts as a global regulator by repressing or promoting the gene expression of several important pathways in *Liberibacter* species. The double approach taken, transcriptome analysis reinforced with the *in silico* methodology, demonstrated complementary results and is therefore a more powerful method to maximize the identification of the LdtR regulon components.

### LdtR binds to the in silico‐predicted sequences in *L. asiaticus*

**Figure 4:**
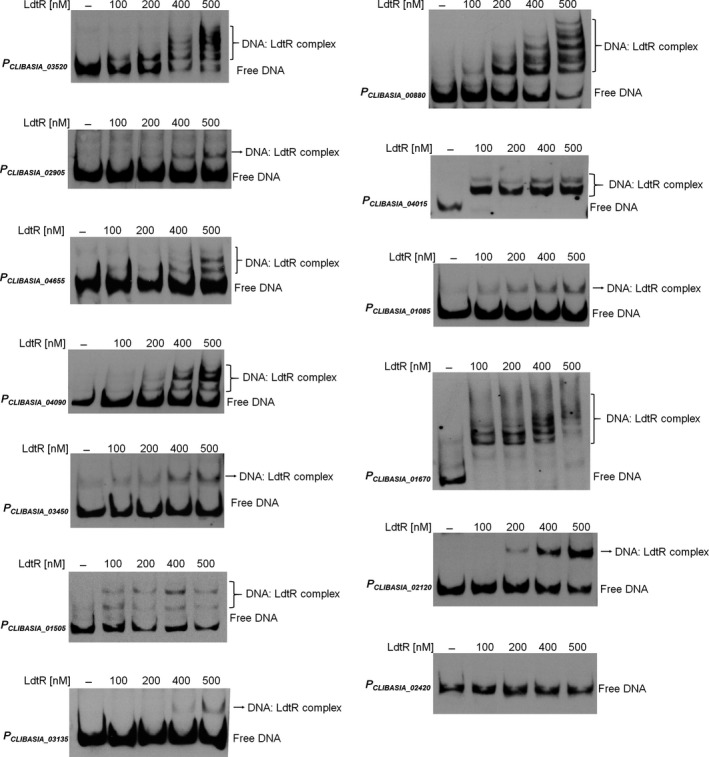
DNA binding assays with LdtR over multiple promoters from *L. asiaticus*. The promoter of *CLIBASIA_02420* (*P*_*CLIBASIA*__*_02420*_) was used as a negative control. The different biotinylated DNA probes were incubated with increasing concentrations of LdtR, as indicated on top of each panel. Protein was not added to the first lane.

The combined analysis of the RNA‐seq data with the *in silico* predictions allowed us to conclude that the LdtR binding sites are largely clustered in two main regions within promoters of *L. asiaticus* (Fig. 3). In order to investigate the significance of the different binding locations, as well as to authenticate the involvement of LdtR in the regulation, a subgroup of genes was selected to perform DNA binding assays. A list of the selected genes from *L. asiaticus* and their homologues in *L. crescens* is shown in Table S3. As such, we selected eight genes with a predicted LdtR binding site located more than 100 bp upstream of the translational start site (−100 to −250 region, Fig. 4). Biotinylated probes were synthesized for the promoters of the following genes: *CLIBASIA_03520*,* CLIBASIA_02905*,* CLIBASIA_04655*,* CLIBASIA_04090*,* CLIBASIA_03450*,* CLIBASIA_01505*,* CLIBASIA_03135* and *CLIBASIA_00880*. Additionally, the promoters of the following genes were selected to test the LdtR binding site located not beyond 100 bp upstream of the translational start site (−100 to +1 region, Fig. 4): *CLIBASIA_04020*,* CLIBASIA_01090*,* CLIBASIA_01670* and *CLIBASIA_02120*. Within this subgroup, the genes *CLIBASIA_01090* and *CLIBASIA_04020* are predicted to form an operon with their corresponding upstream gene; therefore, the promoter regions of *CLIBASIA_01085* and *CLIBASIA_04015* were used in EMSAs. LdtR recognized and bound to all the promoter fragments used in the assays, and the amount of protein used was always in the nanomolar range, indicative of high binding affinity. Of note, the LdtR binding affinity was highest in two of the promoters used in the assays, *P*_*CLIBASIA_04015*_ and *P*_*CLIBASIA_01670*_. The EMSA analysis suggests that the binding affinity of LdtR over those promoters was even higher than the affinity previously described for the promoter of *ldtP* (Pagliai *et al*., 2014). The promoter region of *CLIBASIA_02420*, with no predicted binding sites, was included as negative control. As expected, LdtR did not bind to the *P*_*CLIBASIA_02420*_, even when the protein was increased more than 20 times (Fig. 4). Benzbromarone and phloretin have been previously described as ligands that disrupted the interaction between LdtR and the *ldtP* promoters (Pagliai *et al*., 2014). Consequently, EMSAs were conducted on representative promoters (*P*_*CLIBASIA_02120*_ and *P*_*CLIBASIA_03520*_) to test the effect of these ligands in the binding capabilities of LdtR. It was found that benzbromarone and phloretin disrupted the binding of LdtR to both promoters (Fig. S2). As expected, the promoter of *CLIBASIA_02420* showed no binding of the regulator regardless of the addition of any ligand to the mix (Fig. S2). These *in vitro* results indicate that LdtR might be directly involved in the regulation of gene expression observed earlier *in vivo*.

### The chemical inactivation of LdtR affects the expression of genes in infected orange leaves

**Figure 5:**
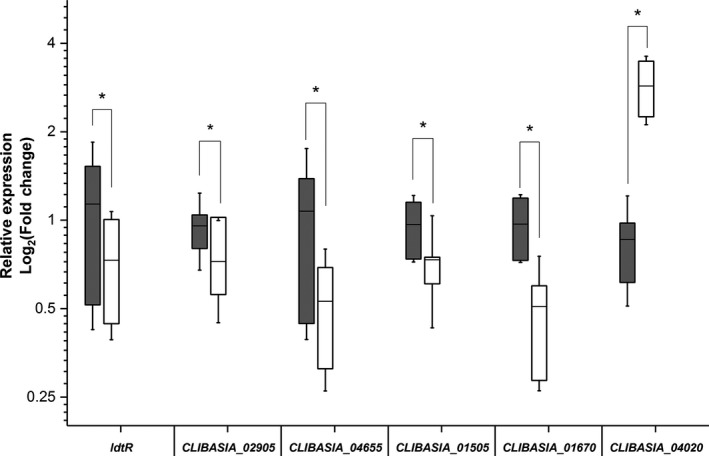
An LdtR ligand modulates the transcriptional activity of LdtR in infected citrus leaves. The expression levels of *ldtR*,*CLIBASIA_02905*,*CLIBASIA_04655*,*CLIBASIA_01505*,*CLIBASIA_01670* and *CLIBASIA_04020* of *L. asiaticus* were assessed to monitor the transcriptional activity of LdtR upon inactivation by benzbromarone. The relative expression values of each gene were obtained by normalizing their levels against the expression levels of 16S rRNA (grey boxes) of *L. asiaticus*. White boxes represent control conditions. The horizontal line in each box plot represents the mean value. The effect of benzbromarone on the transcriptional activity of LdtR was evaluated after 24 h of incubation. The qRT‐PCR data statistical significance was determined using a two‐tailed Student's *t*‐test (*P <* *0.05). The experiments were performed in quadruplicates.

The effect of LdtR inactivation by benzbromarone in a subset of *L. asiaticus* genes was assessed using a model with HLB‐symptomatic *Citrus sinensis* leaves (Pagliai *et al*., 2014). For this study, twelve leaves per experimental condition were collected from highly symptomatic branches and incubated for 24 h in the presence or absence of 1 μM benzbromarone, using DMSO as a control. This concentration of benzbromarone was previously described as subinhibitory for *L. asiaticus*, as the expression of 16S rRNA, *rplJ* and *gyrA* was not modified (Pagliai *et al*., 2014). Our rationale was that at a sublethal concentration of benzbromarone, the expression of genes belonging to the LdtR regulon would be altered, whereas the expression of constitutive genes, such as 16S rRNA and *gyrA* genes will remain constant. The effect of benzbromarone on the expression of *ldtR*,* CLIBASIA_02905*,* CLIBASIA_04655*,* CLIBASIA_01505*,* CLIBASIA_01670* and *CLIBASIA_04020* genes was determined, and their amplification values were normalized against 16S rRNA (Fig. 5). It was found that the presence of benzbromarone resulted in a decreased expression of the transcript levels for *ldtR*,* CLIBASIA_02905*,* CLIBASIA_04655*,* CLIBASIA_01505* and *CLIBASIA_01670* (35%, 24%, 51%, 24% and 47% respectively; *P *<* *0.05). On the other hand, a significant increased expression (330%, *P *<* *0.05) was observed for *CLIBASIA_04020* due to the action of benzbromarone. Similar changes in the gene expression were obtained after normalizing the amplification values against *gyrA* (data not shown). The observed pattern of changes in the gene expression, due to the activity of the LdtR ligand in HLB‐symptomatic leaves, correlated with the results obtained in the *L. crescens* surrogate model (Fig. 2A). Taken together, these results suggest that LdtR performs a dual mechanism of regulation in *L. asiaticus*, acting as a transcriptional activator or repressor depending on the location of the LdtR binding site.

### LdtR binds with high affinity to the promoters of *CLIBASIA_04015* and *CLIBASIA_01670*

**Figure 6:**
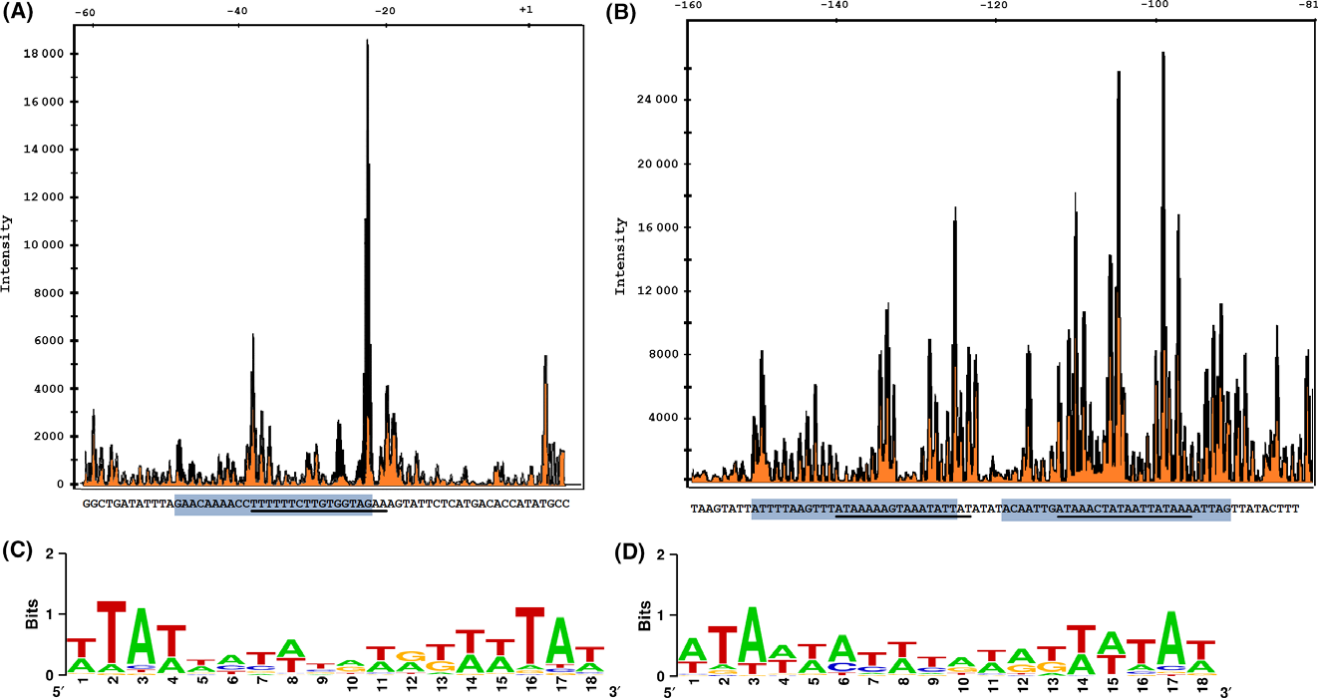
Identification of the LdtR binding site in (A) *P*_*CLIBASIA*__*_04015*_ and (B) *P*_*CLIBASIA*__*_01670*_ promoters. The DNAse I footprinting electropherograms show a fragment of the digested probe in the absence (orange) or presence (black) of LdtR, highlighting the protected region. The LdtR binding box in each promoter is indicated with a light blue box. Underlined is the palindromic sequence identified with RegPredict. The graphical representation (LOGO) of the linear alignment of LdtR binding sites among the homologues in *L. asiaticus* of the (C) up‐regulated or (D) down‐regulated genes identified in the RNA‐seq experiments.

The gel shift assays revealed that LdtR binds with higher affinity to *CLIBASIA_04015* and *CLIBASIA_01670* promoters and more than one DNA:protein complex was observed, suggesting the presence of more than one binding site for LdtR. To determine the characteristics of the LdtR DNA binding site, in both repressor and activator‐regions, a DNase I footprinting assay (Zianni *et al*., 2006) was conducted on the promoter of *CLIBASIA_04015* and *CLIBASIA_01670* respectively. For the promoter of *CLIBASIA_04015*, only one LdtR‐protected box was observed and comprised 30 nucleotides located between −37 and −8, respective to the translational start point (Fig. 6A). In correlation with the observed DNase I protected site, this region contains a LdtR binding site [(−27)TTTTTTCTTGTGGTAGAA(−10)] according to the RegPredict analysis (Fig. 6A). Alternatively, for the promoter of *CLIBASIA_01670*, two protected areas were identified. The first site comprised 31 nucleotides located between −155 and −125 respective to the translational start point (Fig. 6B). According to RegPredict analysis, this protected region encompasses a LdtR binding site [(−141)ATAAAAAGTAAATATTAT(−124)]. The second site comprised 27 nucleotides located between −119 and −93 respective to the translational start point (Fig. 6B). According to RegPredict analysis, this protected region also contains a LdtR binding site [(−112)ATAAACTATAATTATAAA(−95)].

The 182 genes identified in the RNA‐seq with homologues in *L. asiaticus* were gathered depending on their induction or repression profile. This rationale is meant to study the characteristics of the LdtR binding sites among genes where LdtR may act as a repressor (fold change bigger than 1.0 in the RNA‐seq experiments, Table S1) or the sites where LdtR may play a role as an activator (fold change smaller than 1.0 in the RNA‐seq experiments, Table S1). An alignment of the LdtR binding sites from the first group (96 genes up‐regulated by LdtR inactivation) was performed, and a DNA sequence logo (Crooks *et al*., 2004) was generated (Fig. 6C). The same procedure was accomplished with the sites of the genes down‐regulated by LdtR inactivation (86 sequences, Fig. 6D). After comparing the two position weight matrices generated by this approach, it was found the sites have high similarity at the ends of the inverted repeat with distinctive A/T‐rich motif. These observations are positively correlated with the results obtained in the analysis of the *ldtP* promoter, where the crucial residues for the binding of LdtR were located in both ends of the inverted repeat (Pagliai *et al*., 2014). These results suggest that LdtR acts as global transcriptional regulator in *L. asiaticus*, and it does so by recognizing, within the promoters of regulated genes, a DNA inverted repeat motif rich in A/T at both ends.

## Discussion

Many MarR family members have been described as sensors of plant‐ or animal‐derived phenolic compounds (Sulavik *et al*., 1995), suggesting that sensing a phenolic moiety is a common mechanism of regulation by members of this family. LdtR is not an exception as it was demonstrated to bind molecules with a phenolic moiety such as benzbromarone and phloretin (Pagliai *et al*., 2014). In this report, a new feature of the LdtR transcriptional regulator was exposed. Previously described as a transcriptional activator of its own expression as well as *ldtP* (Pagliai *et al*., 2014), we demonstrated that LdtR globally binds in the genome of *L. asiaticus*. LdtR is capable of activating or repressing gene expression from a variety of essential functions, in a mechanism that could be required for the adaptation of the bacterium into their hosts.

A few other cases of MarR family members acting as global regulators have been described. In *Streptomyces roseosporus*, the MarR family transcriptional regulator DptR3 acts as a global regulator for the production of daptomycin and for morphological development. It does so by binding and activating the transcription of the *dpt* gene cluster, involved in daptomycin biosynthesis, and repressing *orf16*, encoding for an ABC transport system involved in the uptake of biosynthetic compounds (Zhang *et al*., 2015). On the other hand, the morphology is thought to be controlled by the binding of DptR3 to the promoters of the *adpA* and *bldD* regulators (Zhang *et al*., 2015). The master virulence regulator in *Salmonella enterica*, SlyA, competes with and counteracts the activity of the general transcriptional silencer H‐NS (Lithgow *et al*., 2007). The binding of SlyA, or its homologue RovA from *Yersinia pestis*, to certain promoters impedes the binding of the H‐NS repressor, which allows transcription or promotes the activation by other regulators, such as PhoP or OmpR (Westermark *et al*., 2000; Ellison and Miller, 2006). It has been recently postulated that SlyA, as well as other global regulators from the MarR family, relies more on the indirect readout and DNA shape than high‐affinity recognition for the DNA (Dolan *et al*., 2011). Some MarR family members may act as both repressors and activators. Possibly, the best‐described example of a dual role for a MarR member is the OhrR protein from *Streptomyces coelicolor*. OhrR is able to bind in the operator region between the divergently oriented genes *ohrR* and *ohrA*, acting as a repressor or activator depending on its oxidation state (Oh *et al*., 2007). Nonetheless, our results suggest this dual behaviour is likely associated with the location of the DNA binding site within the promoters regulated by LdtR. It is also possible that LdtR acts as a global regulator via interactions with other regulatory proteins, similar to the case of PrbP, a transcription accessory protein recently described in *L. asiaticus* (Gardner *et al*., 2016).

To decipher the role of LdtR in the regulation of gene expression in *L. asiaticus,* we performed a double approach. This involved the analysis of the transcript changes due to the presence of LdtR ligands in *L. asiaticus* and the surrogate strain *L. crescens*, as well as an *in silico* prediction of the LdtR binding sites over the entire genome of *L. asiaticus*. Through our strategy, it was discovered that LdtR behaves as a master regulator of gene expression in *L. asiaticus* and is capable of activating or repressing gene expression over a variety of cellular functions. The ability of LdtR to modulate transcription genome‐wide suggests that the bacterium most likely senses changes in the environment (i.e. the transition between the psyllid and the phloem) to activate or repress the expression of certain genes, allowing the bacteria to persist in both hosts. LdtR directly regulates important processes in *L. asiaticus* physiology, such as cell motility (*CLIBASIA_04655*), cell wall biogenesis (*ldtP* and *CLIBASIA_04020*), energy production and conversion (*CLIBASIA_01505*) and transcription (*ldtR* and *CLIBASIA_02905*). LdtR may be indirectly involved in the regulation of other functions, as a putative LdtR box was identified in the promoter of the response regulator *CLIBASIA_03950* (Table S2). A homologue of this response regulator exerts a regulatory role in DNA replication, cell division and morphogenesis in the α‐proteobacteria *Caulobacter crescentus* (Brilli *et al*., 2010). The characteristic asymmetric cell division described in *C. crescentus*,* S. meliloti* and *Agrobacterium tumefaciens* (Hallez *et al*., 2004), suggests that a similar mechanism may be present in *L. asiaticus*. In another example of global regulation, GapR was claimed as a nucleoid‐associated protein in *C. crescentus*. GapR interacts with A/T‐rich sequence promoters of genes involved in the growth and cell cycle differentiation of this bacterium (Ricci *et al*., 2016). Interestingly, a homologue of this newly described DNA binding protein was also identified in our RNA‐seq experiments (*B488_05880*), suggesting LdtR may be involved in the regulation of growth and cell division genes, and perhaps other functions yet to be identified. LdtR likely modulates other processes, such as defence mechanisms, cell cycle control and inorganic ion transport, but further assays are required to determine LdtR's role. In agreement with our data, some of these genes (i.e. *ldtR*,* CLIBASIA_02905* and *CLIBASIA_03135*) have been previously shown to be highly expressed in leaves of infected Valencia sweet orange (*Citrus sinensis*) seedlings, when compared to expression levels observed in the psyllid *Diaphorina citri* (Yan *et al*., 2013).

Among the genes found in the LdtR regulon, some may have an important role in the plant–microbe interaction. A recent study suggested that LotP, encoded by *CLIBASIA_03135*, might play a key role during the plant response to *L. asiaticus* (Loto *et al*., 2017). It is proposed that LotP silences the activity of the GroEL chaperone (Loto *et al*., 2017), which has been described as a MAMP (microbial associated molecular pattern), capable of triggering plant resistance mechanisms (Chaudhary *et al*., 2014). Genes involved in the remodelling of the cell wall, such as the LD‐transpeptidase (*ldtP*) is positively regulated by LdtR; whereas it negatively regulates the alanine and glutamate racemases (encoded by *CLIBASIA_01090* and *CLIBASIA_04020* respectively). The combined activity of LdtP and the glutamate and alanine racemases may change the structure of the peptidoglycan of the bacterium. LD‐transpeptidases catalyse the formation of 3‐3 peptidoglycan cross‐links (Magnet *et al*., 2008). In *Mycobacterium tuberculosis*, mutants of LD‐transpeptidases showed severe differences in the cell size, growth rates and virulence (Schoonmaker *et al*., 2014). Racemases are enzymes that catalyse the production of D‐amino acids from their corresponding L‐enantiomers. In *Vibrio cholerae*, the production of D‐amino acids is required for the remodelling of the peptidoglycan structure in adaptation to stationary phase conditions, resulting in an increased tolerance to osmotic challenge (Lam *et al*., 2009). We hypothesized that *L. asiaticus* may use a similar strategy to evade the plant defence system, leading to the proliferation of the bacterium in the phloem tissue. Some genes with unknown function, such as *CLIBASIA_01670* and *CLIBASIA_00880* were discovered to contain an LdtR binding site, where LdtR most likely acts as an activator. The regulatory role of LdtR over these genes with unknown function may represent another landmark in the myriad of functions regulated by LdtR in *L. asiaticus*.

Our results broadened the current information of how *L. asiaticus* senses fluctuations in the environment and triggers changes in gene expression. In a recent study conducted in *L. crescens*, it was proposed that many of the genes regulated by LdtR reported here (i.e. glutamate racemase, the septum formation protein Maf and the ferredoxin) are essential as no mutants were obtained (Lai *et al*., 2016). It is certainly possible that under laboratory conditions, some genes from *L. crescens* are not essential for the survival of the bacteria, or that the transposon insertion did not render a fully inactive protein. A limitation of our study, using *L. crescens*, is that the expression of genes regulated by LdtR involved in host–microbe interaction may not be identified. However, the combined *in vivo* and *in vitro* analyses performed here highlighted the crucial role of LdtR in the modulation of genes required for the survival of *L. crescens* under laboratory conditions, a process that may be extrapolated to the survival of *L. asiaticus* within its hosts.

## Experimental procedures

### Bacterial strains

*Liberibacter crescens* BT‐1 cells were cultured at 25 °C with moderate agitation (200 rpm) in modified BM7 media as previously described (Pagliai *et al*., 2014). All antibiotics and chemicals were purchased from Sigma‐Aldrich (St. Louis, MO, USA).

### Protein Purification

Protein expression and purification were conducted as previously described (Pagliai *et al*., 2015). Briefly, the H_6X_‐LdtR was overexpressed in *E. coli* BL21‐Star(DE3) cells (Life Technologies, Grand Island, NY, USA). The cells were grown in LB broth with aeration (250 rpm) at 37 °C until OD_600_ = 0.5. LdtR overexpression was induced by adding 0.5 mM IPTG, and the cells were incubated in the shaker at 17 °C overnight. After harvesting, the cells were washed and suspended in binding buffer (500 mM NaCl, 5% glycerol, 50 mM Tris pH 8.0, 5 mM imidazole and 0.5 mM TCEP). Subsequently, the cells were disrupted in a French press and the cell‐free extract loaded in metal chelate affinity column charged with Ni^2+^ (Qiagen, Valencia, CA, USA). The column was washed with binding buffer supplemented with 25 mM imidazole. After elution (binding buffer supplemented with 250 mM imidazole), the purified proteins in solution were dialysed for 16 h in 500 mM NaCl, 5% glycerol, 50 mM Tris pH 8.0 and 0.5 mM TCEP. The concentration of the purified proteins was determined with the Bio‐Rad protein assay, using bovine serum albumin as standard (Bio‐Rad, Hercules, CA, USA).

### Transcriptome analysis

For the mRNA‐seq experiments, *L. crescens* BT‐1 cells were cultured in the presence of phloretin (20 μM), benzbromarone (50 μM) or DMSO as a control (0.05%). The cells were collected by centrifugation at 8000 rpm at 4 °C when the OD_600_ = 0.3 (mid‐exponential phase). Total mRNA was extracted with the RiboPure‐Bacteria (Life Technologies) following the manufacturer's protocol. RNA concentration was determined in a Nanodrop, and their quality was assessed in an Agilent 2100 Bioanalyzer. The rRNA was depleted using the MICROBExpress Bacterial mRNA enrichment kit (Life Technologies) in accordance with the manufacturer's protocol. Single‐end RNA libraries were prepared using the TruSeq Stranded mRNA Library Prep Kit (Illumina, San Diego, CA, USA) followed by sequencing in a HiSeq2500 system. RNA sequencing was performed at the Genome Sciences Facility, Penn State University, Hershey, PA. The assays were performed in duplicates, and approximately 9 million reads were obtained for each analysed library.

The sequencing data were analysed using a pipeline allocated at the HiPerGator supercomputer at University of Florida. Briefly, the raw Sanger sequencing data were modified to trim the Illumina sequencing adapter using Cutadapt (Martin, 2011), followed by sequence processing using the Sickle tool (Joshi and Fass, 2011) to verify the quality of the samples prior to further analyses. Subsequently, all the sequences were mapped against the genome of *L. crescens* BT‐1 using Bowtie2 (Langmead and Salzberg, 2012). At this point, the tRNA and the remaining rRNA sequences were depleted *in silico*. The mapped mRNA sequences were aligned using Samtools (Li *et al*., 2009) and counted using the HTseq tool (Anders *et al*., 2015). The abundance of each transcript from the cells grown in the presence of either phloretin or benzbromarone was compared against the transcripts from cells grown in a DMSO control using the DESeq2 package (Love *et al*., 2014). The full list of the differentially expressed genes is shown in Table S1.

### qRT‐PCR experiments

*L. crescens* BT‐1 cells were cultured in BM7 culture media amended with phloretin (20 μM), benzbromarone (50 μM) or DMSO as a control (0.05%). DMSO was included because phloretin and benzbromarone stock solutions were prepared in DMSO. The cells were collected by centrifugation at 8000 rpm at 4 °C when the OD_600_ = 0.3 (mid‐exponential phase). The total RNAs were extracted with the RiboPure‐Bacteria (Life Technologies) following the manufacturer's protocol.

An *in vitro* model to test chemicals on *L. asiaticus*‐infected leaves (Pagliai *et al*., 2014) was used to assess the role of benzbromarone in the transcriptional activity of LdtR. The RNA extraction from young flushes of highly symptomatic trees, treated with 1 μM benzbromarone or 0.01% DMSO as a control, was conducted as described previously (Gardner *et al*., 2016).

The concentrations of the RNAs were determined using Nanodrop, and the cDNAs were synthesized using the iScript cDNA synthesis kit (Bio‐Rad). qRT‐PCR assays were performed in duplicates for each sample obtained from four independent replicates. The PowerUp SYBR master mix was used as recommended by the manufacturer, and the reactions were carried out in a QuantStudio6 equipment (Life Technologies). The changes of expression (*C*_t_ values) between the samples treated with phloretin or benzbromarone compared to the DMSO control were determined using the $2^{-\Delta \Delta C_{t}}$ method. Amplification of the 16S rRNA or *gyrA* was used as internal control. The primers used during the qRT‐PCR experiments are described in Table S4. The qRT‐PCR plots were generated in MicroCal Origin 9.0 (Northampton, MA, USA).

### Electrophoresis mobility shift assays (EMSAs)

Gel shift assays of LdtR over the selected promoters were carried out as described previously (Pagliai *et al*., 2014). Briefly, fragments of the selected promoters were generated by PCR using biotinylated primers (Table S4). The reaction mix for EMSA contained 1 ng of 5’‐labelled DNA probe, 50 nM Tris‐HCl pH 7.2, 150 mM KCl, 10 mM MgCl_2_, 0.01% Triton X100, 12.5 ng μl^−1^ both Poly(dI‐dC) and Poly(dA‐dT) non‐specific competitor DNAs and purified LdtR (0–600 nM) as properly indicated. The mix was incubated for 20 min at 37 °C, and the electrophoresis was conducted at 4 °C on 6% acrylamide/bisacrylamide non‐denaturing gels in 0.5X Tris‐borate EDTA buffer (TBE) pH 8.3. The DNA was transferred to a Hybond‐N^+^ membrane (GE Healthcare, Pittsburgh, PA, USA) by electroblotting at 250 mA for 45 min in a semidry transfer unit (Fisher Scientific, Pittsburgh, PA, USA) for further detection with the Phototope‐Star Detection Kit (New England Biolabs, Ipswich, MA, USA). The membranes were exposed to Kodak X‐ray films.

### DNase I footprinting

DNase I protection assays were performed on the plus strand by using 5′‐FAM‐labelled probes generated by PCR, using primers CLIB_04015_Fw_FAM and CLIB_04015_Rv for the promoter of *CLIBASIA_04015;* or primers CLIB_01670_Fw_FAM and CLIB_01670_Rv for the promoter of *CLIBASIA_01670* (Table S4). The reaction mixture was the same as used for EMSA assays, except that 7.5 ng μl^−1^ labelled probe, 12 μM LdtR, 0.5 mM CaCl_2_, 2.5 mM MgCl_2_ and 0.025 U of DNase I (New England Biolabs) were added into a 200 μl reaction. The reaction was incubated for 20 min at 37 °C and terminated by incubating 20 min at 70 °C with the addition of 10 mM EDTA, pH 8.0 (Pagliai *et al*., 2014). A digestion reaction without LdtR was included as a control. The digested DNA and sequencing reaction products were analysed at the Plant and Microbe Genomics facility, Ohio State University, Columbus, OH, using a 3730 DNA analyzer, and the protected regions were identified with GeneMapper (Life Technologies), as described earlier (Zianni *et al*., 2006).

### Statistical analyses

The statistical significance of the qRT‐PCR data from *L. crescens* was assessed with an analysis of variance (ANOVA) and a Tukey's HSD *post hoc* test. The statistical significance of the qRT‐PCR data from the citrus‐infected leaves was determined using a paired Student's *t*‐test. Throughout all the assays, a *P*‐value < 0.05 was considered statistically significant, and α = 0.05 was used for the Tukey's HSD testing. The statistical significance was determined over quadruplicate assays.

## Conflict of interest

None declared.

## Supporting information


**Table S1**. List of genes differentially expressed in *L. crescens* treated with 20 µM phloretin.
**Table S2**. The location of the putative LdtR binding sites in *L. asiaticus* genome not identified in the RNA‐seq experiments.
**Table S3**. Annotation of the selected genes for DNA binding assays from *L. asiaticus* and their homologs in *L. crescens*.
**Table S4**. Oligonucleotides used in this study.
**Figure S1**. Correlation between the fold changes of the differentially expressed genes identified in the RNAseq experiments. The linear regression of the fold change values obtained in the benzbromarone and phloretin treatments was calculated in MicroCal Origin 9.0.
**Figure S2**. LdtR ligands disrupt LdtR binding to *P*_*CLIBASIA_02120*_ and *P*_*CLIBASIA_03520*_. EMSAs were conducted in the absence or presence of either benzbromarone or phloretin, as indicated on top of each panel. Protein was not added to the first lane.
