# Supplementary material for: LdtR is a master regulator of gene expression in Liberibacter asiaticus
Source: Microb Biotechnol. 2017 May 15;10(4):896–909. doi: 10.1111/1751-7915.12728 (PMC5481520; doi:10.1111/1751-7915.12728)

Table S1. List of genes differentially expressed in *L. crescens* treated with 20 µM phloretin

| **locus_tag** | **Fold Change** | ***p*adj** | **Blast2Go Annotation** | **COG** |
| --- | --- | --- | --- | --- |
| B488_05980 | 1.715 | 3.11E-27 | UDP-3-O-acyl-N-acetylglucosamine deacetylase | Cell Wall Membrane/Envelope biogenesis |
| B488_07960 | 1.582 | 4.98E-14 | Zinc ABC transporter, periplasmic-binding protein Znu | Inorganic ion transport and metabolism |
| B488_01280 | 1.575 | 4.32E-17 | beta-(1-->2)glucan export ATP-binding/permease NdvA | Defense Mechanisms |
| B488_04740 | 1.456 | 0.000264 | hypothetical protein | no COG |
| B488_05990 | 1.436 | 2.78E-13 | competence lipoprotein ComL | Cell Wall Membrane/Envelope biogenesis |
| B488_01550 | 1.427 | 5.84E-09 | tRNA threonylcarbamoyladenosine biosynthesis protein TsaB | Translation, ribosomal structure and biogenesis |
| B488_05620 | 1.409 | 4.96E-07 | transporter | Transcription |
| B488_11260 | 1.406 | 2.41E-11 | SAM-dependent methyltransferase | Translation, ribosomal structure and biogenesis |
| B488_08910 | 1.403 | 0.001936 | hypothetical protein | no COG |
| B488_13070 | 1.397 | 0.009183 | tryptophan synthase subunit alpha | Amino acid transport and metabolism |
| B488_06000 | 1.383 | 6.64E-09 | DNA recombination protein RecN | Replication, recombination and repair |
| B488_12270 | 1.381 | 3.26E-08 | peptide-methionine (R)-S-oxide reductase | Posttranslational modification, protein turnover, chaperones |
| B488_02580 | 1.377 | 2.94E-07 | NADH-ubiquinone oxidoreductase subunit B | Energy production and conversion |
| B488_11860 | 1.363 | 1.85E-09 | heat-shock protein A | Posttranslational modification, protein turnover, chaperones |
| B488_12920 | 1.346 | 2.28E-08 | pilus assembly protein Flp/PilA | Intracellular trafficking, secretion, and vesicular transport |
| B488_11170 | 1.334 | 3.45E-06 | CDP-diacylglycerol--serine O-phosphatidyltransferase | Lipid transport and metabolism |
| B488_10240 | 1.332 | 0.000428 | hypothetical protein | no COG |
| B488_02660 | 1.329 | 0.000228 | NADH-ubiquinone oxidoreductase subunit J | Energy production and conversion |
| B488_12490 | 1.329 | 8.27E-07 | serine peptidase | Posttranslational modification, protein turnover, chaperones |
| B488_12650 | 1.328 | 7.75E-06 | septation protein A | Cell cycle control |
| B488_07750 | 1.317 | 0.036092 | hypothetical protein | no COG |
| B488_13280 | 1.315 | 1.60E-06 | 3-oxoacyl-ACP synthase | Lipid transport and metabolism |
| B488_02620 | 1.314 | 2.33E-06 | NADH-quinone oxidoreductase subunit F | Energy production and conversion |
| B488_07780 | 1.313 | 0.035547 | hypothetical protein | no COG |
| B488_00970 | 1.309 | 0.000555 | threonine synthase | Amino acid transport and metabolism |
| B488_10780 | 1.303 | 4.75E-07 | choline ABC transporter permease subunit | Amino acid transport and metabolism |
| B488_02640 | 1.298 | 2.64E-05 | NADH-quinone oxidoreductase subunit H | Energy production and conversion |
| B488_00450 | 1.296 | 0.000351 | phosphatidate cytidylyltransferase | Lipid transport and metabolism |
| B488_RS03805 | 1.295 | 0.027739 | ABC transporter | Inorganic ion transport and metabolism |
| B488_08070 | 1.286 | 0.002245 | hypothetical protein | no COG |
| B488_04950 | 1.285 | 0.02763 | hypothetical protein | no COG |
| B488_13290 | 1.285 | 3.05E-07 | organic hydroperoxide resistance protein | Defense Mechanisms |
| B488_01470 | 1.282 | 0.002418 | hypothetical protein | no COG |
| B488_11510 | 1.28 | 5.39E-08 | outer-membrane immunogenic protein | Cell Wall Membrane/Envelope biogenesis |
| B488_07830 | 1.272 | 0.038749 | portal protein | no COG |
| B488_13120 | 1.271 | 0.014693 | anthranilate synthase component I | Coenzyme transport and metabolism |
| B488_04190 | 1.269 | 0.000273 | 30S ribosomal protein S17 | Translation, ribosomal structure and biogenesis |
| B488_12890 | 1.269 | 0.000121 | pilus assembly protein Flp/PilA | Intracellular trafficking, secretion, and vesicular transport |
| B488_RS05980 | 1.267 | 0.00414 | camphor resistance protein CrcB | Cell cycle control |
| B488_RS03820 | 1.267 | 0.003354 | PhnP | Inorganic ion transport and metabolism |
| B488_07950 | 1.264 | 0.044506 | zinc ABC transporter inner membrane permease ZnuB | Inorganic ion transport and metabolism |
| B488_08360 | 1.264 | 0.010271 | glycine oxidase | Amino acid transport and metabolism |
| B488_11630 | 1.262 | 2.26E-06 | hypothetical protein | no COG |
| B488_01190 | 1.259 | 0.000419 | Putative Zn-dependent protease | General function prediction only |
| B488_03200 | 1.259 | 0.000175 | 30S ribosomal protein S18 | Translation, ribosomal structure and biogenesis |
| B488_02650 | 1.252 | 0.003025 | NADH-quinone oxidoreductase subunit I | Energy production and conversion |
| B488_00270 | 1.247 | 0.00626 | Putative flippase GtrA (transmembrane translocase of bactoprenol-linked glucose) | Lipid transport and metabolism |
| B488_07430 | 1.246 | 6.34E-05 | succinate--CoA ligase subunit alpha | Energy production and conversion |
| B488_05260 | 1.246 | 1.12E-05 | 30S ribosomal protein S4 | Translation, ribosomal structure and biogenesis |
| B488_08470 | 1.242 | 2.50E-05 | 50S ribosomal protein L1 | Translation, ribosomal structure and biogenesis |
| B488_08490 | 1.241 | 0.000159 | transcription termination/antitermination protein NusG | Transcription |
| B488_02590 | 1.24 | 0.012001 | NADH dehydrogenase | Energy production and conversion |
| B488_03490 | 1.24 | 0.000467 | tolB protein precursor | Intracellular trafficking, secretion, and vesicular transport |
| B488_05610 | 1.233 | 0.003054 | fucose/glucose/galactose-like permease | Carbohydrate transport and metabolism |
| B488_00040 | 1.228 | 0.005426 | chromosome partitioning protein ParB | Cell cycle control |
| B488_RS01785 | 1.224 | 8.79E-05 | hypothetical protein | no COG |
| B488_RS01535 | 1.223 | 0.003025 | hypothetical protein | no COG |
| B488_RS01535 | 1.22 | 0.021449 | cytochrome P450 hydroxylase | Defense Mechanisms |
| B488_RS06085 | 1.219 | 0.017663 | hypothetical protein | no COG |
| B488_10770 | 1.219 | 0.001965 | choline ABC transporter ATP-binding protein | Amino acid transport and metabolism |
| B488_05250 | 1.218 | 0.048003 | glutamate racemase | Cell Wall Membrane/Envelope biogenesis |
| B488_RS02415 | 1.218 | 0.040985 | hypothetical protein | no COG |
| B488_13420 | 1.217 | 0.001618 | hypothetical protein | no COG |
| B488_00570 | 1.217 | 0.000624 | DNA polymerase III subunit gamma/tau | Replication, recombination and repair |
| B488_03210 | 1.215 | 0.000158 | 30S ribosomal protein S6 | Translation, ribosomal structure and biogenesis |
| B488_09320 | 1.212 | 0.010499 | ferredoxin | no COG |
| B488_01540 | 1.212 | 0.000274 | Fe-S cluster biogenesis protein NfuA, 4Fe-4S-binding domain | Posttranslational modification, protein turnover, chaperones |
| B488_07190 | 1.21 | 0.001238 | 16S rRNA (cytosine(1402)-N(4))-methyltransferase | Translation, ribosomal structure and biogenesis |
| B488_07500 | 1.208 | 0.023527 | succinate dehydrogenase hydrophobic membrane anchor protein | Energy production and conversion |
| B488_00650 | 1.208 | 0.001438 | hypothetical protein | no COG |
| B488_10380 | 1.204 | 0.000932 | IMP dehydrogenase | Nucleotide transport and metabolism |
| B488_07450 | 1.203 | 0.004811 | malate dehydrogenase | Energy production and conversion |
| B488_02900 | 1.2 | 0.00414 | two-component sensor histidine kinase | Signal transduction mechanisms |
| B488_09310 | 1.199 | 0.004508 | iron permease | Inorganic ion transport and metabolism |
| B488_RS01340 | 1.199 | 0.002693 | NADH:ubiquinone oxidoreductase subunit L | Energy production and conversion |
| B488_10100 | 1.197 | 0.001347 | beta-ketoacyl | Lipid transport and metabolism |
| B488_11910 | 1.195 | 0.000281 | 30S ribosomal protein S20 | Translation, ribosomal structure and biogenesis |
| B488_03670 | 1.193 | 0.024666 | ribonuclease HII | Replication, recombination and repair |
| B488_08800 | 1.192 | 0.027739 | hydrolase | General function prediction only |
| B488_03500 | 1.19 | 0.002972 | peptidoglycan-associated lipoprotein | Cell Wall Membrane/Envelope biogenesis |
| B488_02800 | 1.189 | 0.025851 | protein-(glutamine-N5) methyltransferase, release factor-specific\"" | Translation, ribosomal structure and biogenesis |
| B488_07460 | 1.189 | 0.011854 | ATPase | General function prediction only |
| B488_09960 | 1.186 | 0.003025 | sodium:proton antiporter | Inorganic ion transport and metabolism |
| B488_03460 | 1.183 | 0.00298 | protein TolQ | Intracellular trafficking, secretion, and vesicular transport |
| B488_12420 | 1.182 | 0.018264 | diacylglyceryl transferase | Cell Wall Membrane/Envelope biogenesis |
| B488_10280 | 1.181 | 0.012047 | Branched-chain amino acid transport system permease LivM | Amino acid transport and metabolism |
| B488_01160 | 1.177 | 0.033856 | orotidine 5\'-phosphate decarboxylase | Nucleotide transport and metabolism |
| B488_12570 | 1.176 | 0.006455 | two-component sensor histidine kinase | Signal transduction mechanisms |
| B488_07470 | 1.175 | 0.006247 | flagellar hook-length control protein FliK | Cell Motility |
| B488_02630 | 1.175 | 0.003675 | NADH-quinone oxidoreductase subunit G | Energy production and conversion |
| B488_06580 | 1.174 | 0.004762 | Outer membrane protein OmpA | Cell Wall Membrane/Envelope biogenesis |
| B488_06780 | 1.173 | 0.023527 | glycosyl transferase | General function prediction only |
| B488_00480 | 1.172 | 0.030729 | UDP-3-O-(3-hydroxymyristoyl)glucosamine N-acyltransferase | Cell Wall Membrane/Envelope biogenesis |
| B488_12940 | 1.172 | 0.026324 | hypothetical protein | Function unknown |
| B488_12510 | 1.17 | 0.029323 | sensor histidine kinase | Signal transduction mechanisms |
| B488_02910 | 1.169 | 0.017663 | sigma-54-dependent Fis family transcriptional regulator | Signal transduction mechanisms |
| B488_04960 | 1.168 | 0.037934 | hypothetical protein | no COG |
| B488_02600 | 1.166 | 0.016748 | NADH dehydrogenase subunit D | Energy production and conversion |
| B488_09600 | 1.165 | 0.028127 | LysR family transcriptional regulator | Transcription |
| B488_12860 | 1.163 | 0.024666 | prepilin peptidase CpaA | Posttranslational modification, protein turnover, chaperones |
| B488_00820 | 1.163 | 0.009744 | cytochrome ubiquinol oxidase subunit I | Energy production and conversion |
| B488_12630 | 1.162 | 0.013493 | phosphate ABC transporter permease | Inorganic ion transport and metabolism |
| B488_10120 | 1.161 | 0.007219 | beta-ketoacyl-ACP reductase | Lipid transport and metabolism |
| B488_07350 | 1.159 | 0.032308 | F0F1 ATP synthase subunit A | Energy production and conversion |
| B488_01350 | 1.158 | 0.040488 | GntR family transcriptional regulator | Transcription |
| B488_06010 | 1.157 | 0.028507 | DNA ligase (NAD(+)) LigA | Replication, recombination and repair |
| B488_03660 | 1.157 | 0.019969 | cysteine protease | no COG |
| B488_06970 | 1.156 | 0.048225 | dihydrodipicolinate reductase | Amino acid transport and metabolism |
| B488_11950 | 1.155 | 0.032934 | non-canonical purine NTP pyrophosphatase | Nucleotide transport and metabolism |
| B488_00400 | 1.155 | 0.00593 | 30S ribosomal protein S2 | Translation, ribosomal structure and biogenesis |
| B488_01450 | 1.154 | 0.03024 | cytochrome d ubiquinol oxidase subunit II | Energy production and conversion |
| B488_05270 | 1.152 | 0.024 | glutaminase | Amino acid transport and metabolism |
| B488_02860 | 1.15 | 0.030065 | hypothetical protein | General function prediction only |
| B488_03380 | 1.15 | 0.010416 | peptidase S41 | Posttranslational modification, protein turnover, chaperones |
| B488_04260 | 1.149 | 0.040396 | 50S ribosomal protein L18 | Translation, ribosomal structure and biogenesis |
| B488_07200 | 1.148 | 0.017663 | cell division/cell wall cluster transcriptional repressor MraZ | Translation, ribosomal structure and biogenesis |
| B488_01060 | 1.147 | 0.022422 | enoyl-[acyl-carrier-protein] reductase | Lipid transport and metabolism |
| B488_09150 | 1.144 | 0.015226 | ATP-dependent protease ATP-binding subunit ClpX | Posttranslational modification, protein turnover, chaperones |
| B488_04290 | 1.143 | 0.031953 | 50S ribosomal protein L15 | Translation, ribosomal structure and biogenesis |
| B488_00470 | 1.141 | 0.006824 | outer membrane protein assembly factor BamA | Cell Wall Membrane/Envelope biogenesis |
| B488_08740 | 1.14 | 0.020911 | Fe-S cluster assembly protein SufB | Posttranslational modification, protein turnover, chaperones |
| B488_03480 | 1.139 | 0.021449 | cell envelope integrity/translocation protein TolA | Cell Wall Membrane/Envelope biogenesis |
| B488_08770 | 1.134 | 0.02574 | ATP-dependent Clp protease ATP-binding subunit ClpA | Posttranslational modification, protein turnover, chaperones |
| B488_11600 | 1.134 | 0.020507 | GTPase HflX | Translation, ribosomal structure and biogenesis |
| B488_04250 | 1.131 | 0.031862 | 50S ribosomal protein L6 | Translation, ribosomal structure and biogenesis |
| B488_07440 | 1.13 | 0.031085 | succinate--CoA ligase subunit beta | Energy production and conversion |
| B488_13350 | 1.13 | 0.028442 | RNA polymerase sigma factor RpoD | Transcription |
| B488_05970 | 1.128 | 0.031421 | cell division protein FtsZ | Cell cycle control |
| B488_03530 | 1.127 | 0.029323 | hypothetical protein | Posttranslational modification, protein turnover, chaperones |
| B488_10950 | 1.123 | 0.031074 | citrate (Si)-synthase | Energy production and conversion |
| B488_13370 | 1.122 | 0.033856 | RND efflux transporter | Defense Mechanisms |
| B488_10250 | 0.892 | 0.030136 | trigger factor | Posttranslational modification, protein turnover, chaperones |
| B488_10110 | 0.884 | 0.044635 | acyl carrier protein | Lipid transport and metabolism |
| B488_11030 | 0.884 | 0.040488 | pyruvate dehydrogenase complex dihydrolipoamide acetyltransferase | Energy production and conversion |
| B488_00800 | 0.881 | 0.049316 | 50S ribosomal protein L33 | Translation, ribosomal structure and biogenesis |
| B488_02850 | 0.881 | 0.023527 | Superoxide dismutase (Fe) | Inorganic ion transport and metabolism |
| B488_01760 | 0.88 | 0.036531 | sensor histidine kinase | Signal transduction mechanisms |
| B488_06460 | 0.88 | 0.02387 | threonine--tRNA ligase | Translation, ribosomal structure and biogenesis |
| B488_06440 | 0.88 | 0.007114 | kinesin-like protein | Cell Motility |
| B488_00360 | 0.878 | 0.029681 | transcriptional regulator | Transcription |
| B488_12590 | 0.876 | 0.019969 | nucleotide exchange factor GrpE | Posttranslational modification, protein turnover, chaperones |
| B488_13210 | 0.875 | 0.021122 | membrane protein insertase YidC | Cell Wall Membrane/Envelope biogenesis |
| B488_RS06290 | 0.874 | 0.037459 | hypothetical protein | no COG |
| B488_00430 | 0.874 | 0.036515 | ribosome-recycling factor | Translation, ribosomal structure and biogenesis |
| B488_03700 | 0.872 | 0.027366 | hypothetical protein | no COG |
| B488_10370 | 0.872 | 0.007128 | carboxypeptidase-like protein | Amino acid transport and metabolism |
| B488_01090 | 0.872 | 0.005749 | hypothetical protein | Function unknown |
| B488_13390 | 0.87 | 0.030729 | Co/Zn/Cd efflux system membrane fusion protein | Cell Wall Membrane/Envelope biogenesis |
| B488_04600 | 0.869 | 0.029323 | DNA topoisomerase IV subunit B | Replication, recombination and repair |
| B488_RS00375 | 0.869 | 0.004114 | ribonuclease G | Translation, ribosomal structure and biogenesis |
| B488_01790 | 0.865 | 0.039617 | helix-turn-helix transcriptional regulator | Transcription |
| B488_06830 | 0.862 | 0.031074 | ATP-dependent DNA helicase RecG | Replication, recombination and repair |
| B488_01570 | 0.861 | 0.016348 | tRNA (N6-isopentenyl adenosine(37)-C2)-methylthiotransferase MiaB | Translation, ribosomal structure and biogenesis |
| B488_11820 | 0.861 | 0.008035 | phage lysin glycosyl hydrolase family 25 | Cell Wall Membrane/Envelope biogenesis |
| B488_00080 | 0.86 | 0.039291 | YggS family pyridoxal phosphate enzyme | General function prediction only |
| B488_08870 | 0.858 | 0.021796 | hypothetical protein | no COG |
| B488_11080 | 0.858 | 0.013839 | protein translocase TatA | Intracellular trafficking, secretion, and vesicular transport |
| B488_06340 | 0.856 | 0.02415 | thymidylate synthase | Nucleotide transport and metabolism |
| B488_05300 | 0.855 | 0.027987 | YrbA protein | Signal transduction mechanisms |
| B488_00580 | 0.853 | 0.015545 | hypothetical protein | General function prediction only |
| B488_01730 | 0.85 | 0.047715 | ferredoxin | Energy production and conversion |
| B488_05320 | 0.848 | 0.005472 | lipoprotein | no COG |
| B488_06480 | 0.846 | 0.015713 | hypothetical protein | no COG |
| B488_01810 | 0.845 | 0.030819 | flagellar biosynthesis protein FlhB | Cell Motility |
| B488_12830 | 0.844 | 0.000894 | hypothetical protein | Extracellular structures |
| B488_13470 | 0.839 | 0.001408 | ATP-dependent protease ATP-binding subunit HslU | Posttranslational modification, protein turnover, chaperones |
| B488_08630 | 0.838 | 0.031952 | 5-(carboxyamino)imidazole ribonucleotide mutase | Nucleotide transport and metabolism |
| B488_13020 | 0.837 | 0.047444 | hypothetical protein | Function unknown |
| B488_03930 | 0.835 | 0.004522 | glucose-6-phosphate dehydrogenase | Carbohydrate transport and metabolism |
| B488_05310 | 0.834 | 0.001569 | phosphoribosylformylglycinamidine synthase II | Nucleotide transport and metabolism |
| B488_08180 | 0.833 | 0.04272 | phosphoribosylamine--glycine ligase | Nucleotide transport and metabolism |
| B488_03760 | 0.833 | 0.021464 | endonuclease III | Replication, recombination and repair |
| B488_01600 | 0.833 | 0.003025 | apolipoprotein N-acyltransferase | Cell Wall Membrane/Envelope biogenesis |
| B488_13190 | 0.833 | 0.001074 | 50S ribosomal protein L34 | no COG |
| B488_08150 | 0.832 | 0.018924 | exodeoxyribonuclease V subunit alpha | no COG |
| B488_12430 | 0.831 | 0.003675 | hypothetical protein | Function unknown |
| B488_13150 | 0.829 | 0.023469 | hypothetical protein | Function unknown |
| B488_11040 | 0.829 | 0.00032 | dihydrolipoamide dehydrogenase | Energy production and conversion |
| B488_00390 | 0.826 | 0.021122 | bis(5\'-nucleosyl)-tetraphosphatase | General function prediction only |
| B488_13610 | 0.825 | 0.001911 | two-component sensor histidine kinase | no COG |
| B488_01880 | 0.824 | 0.049316 | hypothetical protein | Nucleotide transport and metabolism |
| B488_11130 | 0.824 | 0.005082 | lipoprotein | Cell Wall Membrane/Envelope biogenesis |
| B488_05880 | 0.823 | 0.034347 | hypothetical protein | no COG |
| B488_00910 | 0.821 | 0.041883 | exodeoxyribonuclease VII small subunit | Replication, recombination and repair |
| B488_04970 | 0.818 | 0.001539 | hypothetical protein | no COG |
| B488_04380 | 0.816 | 0.017918 | type I methionyl aminopeptidase | Translation, ribosomal structure and biogenesis |
| B488_12100 | 0.816 | 0.000207 | nicotinate-nicotinamide nucleotide adenylyltransferase | Coenzyme transport and metabolism |
| B488_13590 | 0.814 | 0.015547 | PTS permease (IIAMan), nitrogen regulatory IIA protein | Carbohydrate transport and metabolism |
| B488_09130 | 0.811 | 0.000178 | DNA-binding protein HU-beta | Replication, recombination and repair |
| B488_01010 | 0.811 | 0.000121 | hemagglutinin protein | Signal transduction mechanisms |
| B488_11000 | 0.81 | 0.004965 | septum formation initiator protein | Cell cycle control |
| B488_10920 | 0.808 | 0.023527 | hypothetical protein | no COG |
| B488_09490 | 0.808 | 0.010271 | chemotaxis MotD protein | no COG |
| B488_05400 | 0.807 | 0.034147 | hypothetical protein | no COG |
| B488_12050 | 0.805 | 0.000602 | 50S ribosomal protein L21 | Translation, ribosomal structure and biogenesis |
| B488_00230 | 0.804 | 0.000366 | aminopeptidase S | Amino acid transport and metabolism |
| B488_12470 | 0.804 | 8.58E-05 | hypothetical protein | Cell Wall Membrane/Envelope biogenesis |
| B488_02260 | 0.802 | 0.000813 | tRNA-specific adenosine deaminase | Translation, ribosomal structure and biogenesis |
| B488_00940 | 0.802 | 0.000263 | restriction endonuclease subunit M | Replication, recombination and repair |
| B488_13740 | 0.801 | 0.00298 | shikimate dehydrogenase | Amino acid transport and metabolism |
| B488_08990 | 0.8 | 0.009612 | acyltransferase | Cell Wall Membrane/Envelope biogenesis |
| B488_04360 | 0.799 | 0.003342 | hypothetical protein | no COG |
| B488_RS05705 | 0.799 | 3.23E-05 | hypothetical protein | no COG |
| B488_RS03075 | 0.797 | 0.000121 | rare lipoprotein A | Cell Wall Membrane/Envelope biogenesis |
| B488_00930 | 0.795 | 0.002245 | rRNA (cytidine-2\'-O-)-methyltransferase | Translation, ribosomal structure and biogenesis |
| B488_01300 | 0.795 | 0.000175 | transcription elongation factor GreA | Transcription |
| B488_09380 | 0.794 | 0.02574 | flagellar biosynthetic protein FliR | Cell Motility |
| B488_10300 | 0.794 | 0.001211 | hypothetical protein | no COG |
| B488_09860 | 0.793 | 0.003127 | secretion protein HlyD | Cell Wall Membrane/Envelope biogenesis |
| B488_08810 | 0.792 | 0.034147 | hypothetical protein | Function unknown |
| B488_08080 | 0.792 | 0.000145 | hypothetical protein | no COG |
| B488_RS01120 | 0.789 | 1.12E-05 | ribosomal large subunit pseudouridine synthase B | Translation, ribosomal structure and biogenesis |
| B488_13650 | 0.788 | 0.002709 | thiol reductase thioredoxin | no COG |
| B488_07300 | 0.785 | 7.48E-06 | Bipolar DNA helicase | Replication, recombination and repair |
| B488_09170 | 0.783 | 2.05E-06 | 50S ribosomal protein L13 | Translation, ribosomal structure and biogenesis |
| B488_13490 | 0.78 | 0.000814 | imidazoleglycerol-phosphate dehydratase | Amino acid transport and metabolism |
| B488_02400 | 0.777 | 0.000358 | periplasmic chaperone for outer membrane proteins SurA | Posttranslational modification, protein turnover, chaperones |
| B488_04790 | 0.775 | 0.045982 | hypothetical protein | no COG |
| B488_05760 | 0.775 | 3.65E-05 | hypothetical protein | no COG |
| B488_10390 | 0.771 | 0.001243 | Predicted transglutaminase-like cysteine proteinase | Posttranslational modification, protein turnover, chaperones |
| B488_03340 | 0.77 | 0.001239 | hypothetical protein | no COG |
| B488_08500 | 0.769 | 1.99E-06 | preprotein translocase subunit SecE | Intracellular trafficking, secretion, and vesicular transport |
| B488_08960 | 0.768 | 9.17E-05 | hypothetical protein | no COG |
| B488_13480 | 0.768 | 9.72E-06 | ATP-dependent protease subunit HslV | Posttranslational modification, protein turnover, chaperones |
| B488_07620 | 0.76 | 0.001271 | queuosine biosynthesis protein QueD | Coenzyme transport and metabolism |
| B488_13750 | 0.759 | 0.000742 | septum formation inhibitor Maf | Cell Wall Membrane/Envelope biogenesis |
| B488_13220 | 0.757 | 4.29E-05 | YihA family ribosome biogenesis GTP-binding protein | Cell cycle control |
| B488_08650 | 0.756 | 3.26E-05 | 50S ribosomal protein L36 | Translation, ribosomal structure and biogenesis |
| B488_04230 | 0.746 | 2.94E-07 | 30S ribosomal protein S14 | Translation, ribosomal structure and biogenesis |
| B488_04180 | 0.744 | 0.000508 | 50S ribosomal protein L29 | Translation, ribosomal structure and biogenesis |
| B488_00870 | 0.74 | 2.58E-06 | translation initiation factor IF-1 | Translation, ribosomal structure and biogenesis |
| B488_07600 | 0.737 | 4.56E-06 | transmembrane protein | Cell Wall Membrane/Envelope biogenesis |
| B488_04580 | 0.733 | 5.63E-07 | hypothetical protein | Function unknown |
| B488_09010 | 0.73 | 6.44E-08 | hypothetical protein | no COG |
| B488_12000 | 0.729 | 0.000624 | tryptophan-rich sensory protein | Signal transduction mechanisms |
| B488_08270 | 0.727 | 7.48E-06 | hypothetical protein | no COG |
| B488_09940 | 0.726 | 0.001034 | hypothetical protein | no COG |
| B488_10150 | 0.722 | 0.006914 | manganese/iron transporter ATP-binding protein | Inorganic ion transport and metabolism |
| B488_06350 | 0.721 | 4.73E-07 | dihydrofolate reductase | Coenzyme transport and metabolism |
| B488_01640 | 0.721 | 2.74E-07 | hypothetical protein | Function unknown |
| B488_03190 | 0.704 | 3.61E-08 | hypothetical protein | Cell Wall Membrane/Envelope biogenesis |
| B488_08200 | 0.702 | 8.00E-05 | hypothetical protein | no COG |
| B488_03870 | 0.697 | 2.74E-07 | hypothetical protein | Cell Motility |
| B488_10140 | 0.687 | 0.001643 | Manganese ABC transporter substrate-binding protein SitA | Inorganic ion transport and metabolism |
| B488_07580 | 0.683 | 6.89E-06 | hypothetical protein | Cell Wall Membrane/Envelope biogenesis |
| B488_RS02260 | 0.677 | 0.001438 | hypothetical protein | no COG |
| B488_10170 | 0.657 | 2.22E-07 | Manganese ABC transporter inner membrane permease SitD | Inorganic ion transport and metabolism |
| B488_10160 | 0.611 | 2.64E-05 | Manganese ABC transporter inner membrane permease SitC | Inorganic ion transport and metabolism |
| B488_12120 | 0.574 | 1.05E-17 | ABC transporter ATP-binding protein | Defense Mechanisms |
| B488_01800 | 0.574 | 5.16E-18 | helix-turn-helix transcriptional regulator | Transcription |
| B488_04080 | 0.528 | 2.68E-09 | translation elongation factor Tu | Translation, ribosomal structure and biogenesis |
| B488_RS05770 | 0.123 | 1.88E-177 | hypothetical protein | Replication, recombination and repair |

Table S2. The location of the putative LdtR binding sites in *L. asiaticus* genome not identified in the RNA-seq experiments.

| ***L. asiaticus***  **locus_tag** | **Position^1^** | **Sequence** | **Blast2Go Annotation** | ***L. crescens* locus_tag** |
| --- | --- | --- | --- | --- |
| CLIBASIA_00075 | -159 | ATAATACTCGAAATATAG | Ribonucleoside-diphosphate reductase subunit beta | not found |
| CLIBASIA_00105 | -85 | TTAATAAATGTGAATTAA | RNA polymerase subunit beta | B488_08430 |
| CLIBASIA_00600 | -198 | ATAAACTATTTGGTATAA | Hypothetical Protein | not found |
| CLIBASIA_00880 | -109 | TTAATAAAAAAATTTTAT | Metallophosphatase | B488_03970 |
| CLIBASIA_00905 | -114 | ATATTATTTATAGATCAT | Methionyl-tRNA formyltransferase | B488_13310 |
| CLIBASIA_00910 | -175 | TTTTTCCATATGGTATAT | Peptide deformylase | B488_13320 |
| CLIBASIA_00940 | -44 | TTAAAAAATAAATTTTAT | Hypothetical Protein | not found |
| CLIBASIA_01085 | -173 | ATAATAAATAAGGAAACA | DNA Helicase | B488_03170 |
| CLIBASIA_01155 | 10 | TCATTCCTCATGTTTTAA | Lumazine synthase | B488_10850 |
| CLIBASIA_01365 | -78 | TTATAATATTTATTTTAT | Hypothetical Protein | B488_06480 |
| CLIBASIA_01435 | 19 | GTATTCTTTTAGGAATAT | 3-deoxy-D-manno-octulosonic acid transferase | B488_02330 |
| CLIBASIA_01445 | -24 | ATAATAAATGAGATTTAT | (2 ),5 -bisphosphate nucleotidase | B488_02300 |
| CLIBASIA_01520 | -111 | ATATTATAAAAGTTGTAT | Aspartate tRNA ligase | B488_01700 |
| CLIBASIA_01715 | -134 | TTATTATAAGGATTATAA | 5-enolpyruvylshikimate-3-phosphate synthase | B488_01100 |
| CLIBASIA_01925 | -56 | ATAATAAATATCGTATAT | Phosphate acyltransferase | B488_03630 |
| CLIBASIA_02055 | -34 | TTAAAAGATAATTTTTAT | Flagellar hook associated protein | B488_09450 |
| CLIBASIA_02300 | -91 | ATATAATAAAAGGAATAA | Cysteine-tRNA ligase | B488_03110 |
| CLIBASIA_02330 | -103 | CTAGACCTTATAGTATAA | Uncharacterzed protein | B488_11210 |
| CLIBASIA_02380 | -221 | CTATAATTGATGGAATAA | L,D transpeptidase YafK | B488_11360 |
| CLIBASIA_02385 | -153 | ATACTATTTAATGATTAT | Hypothetical Protein | B488_11380 |
| CLIBASIA_02480 | -58 | CTAAAAATCATAGTTTAA | Hypothetical Protein | B488_11690 |
| CLIBASIA_02595 | -31 | TTATTATTTAAAGGGTAT | Hypothetical Protein | not found |
| CLIBASIA_02610 | -7 | TTAATTTATGTATTTTAT | Iron-regulated protein A | not found |
| CLIBASIA_02750 | -88 | TTATTACTTATGGAATAG | Carbonic anhydrase | B488_12310 |
| CLIBASIA_02795 | -95 | TTATAAAAGAAAGTATAT | Pyruvate dehydrogenase E1 component subunit alpha | B488_11010 |
| CLIBASIA_02830 | -167 | TTTTTATATATATAATAA | Hypothetical Protein | not found |
| CLIBASIA_02835 | -78 | TTAAAATATCTATGATAT | Serine tRNA ligase | B488_11110 |
| CLIBASIA_02895 | -42 | ATAATACTAAGAGATTAA | Hypothetical Protein | not found |
| CLIBASIA_03085 | -63 | TTATATTCCGAGGTATAT | Hypothetical Protein | B488_12880 |
| CLIBASIA_03210 | -85 | ATATCACATTAGTAATAA | Polyprenyl synthetase | B488_00690 |
| CLIBASIA_03450 | -163 | ATCCACTTTATATTTTCA | DNA translocase | B488_00110 |
| CLIBASIA_03505 | -21 | ATAATCTAGGTGGTTTAC | Transcription terminator factor Rho | not found |
| CLIBASIA_03645 | -104 | GTAATACATAATGAATAA | DNA primase | B488_05750 |
| CLIBASIA_03650 | 8 | TTATTTTTCGCATTTTAA | Uncharacterized phosphosugar isomerase | B488_05770 |
| CLIBASIA_03715 | -36 | TTATTATATTAGTTTTAC | GroES | B488_02550 |
| CLIBASIA_03855 | -130 | TTATTAGTTGAGGGATAA | Peptide chain release factor 1 | B488_02790 |
| CLIBASIA_03900 | -153 | TTAATCCTTTGAGATTAA | Membrane insertion efficiency factor | not found |
| CLIBASIA_03950 | -148 | TTATAACTGGTATACTAA | Cell cycle response regulator | B488_10320 |
| CLIBASIA_03955 | -74 | TTAATTTTTTTATATTAA | Hypothetical Protein | B488_10310 |
| CLIBASIA_04090 | -129 | ATAATTTATATTTATAAT | dTDP-4-dehydrorhamnose 3,5-epimerase | B488_10000 |
| CLIBASIA_04410 | -168 | TTAATCTTTAACCTTTAA | Hypothetical Protein | not found |
| CLIBASIA_04535 | -75 | ATATACCTTGTAAAATAT | Ribonucleoside-diphosphate reductase | not found |
| CLIBASIA_04540 | -102 | ATATTTTACAAGGTATAT | Hypothetical Protein | not found |
| CLIBASIA_04875 | -107 | CTATTCTACAAAGATTAA | Oxygen-dependent coproporphyrinogen | B488_08410 |
| CLIBASIA_04905 | -129 | TTAGTACTTTAATATTAT | Diguanylate cyclase | B488_05930 |
| CLIBASIA_05010 | -76 | TTATTACTTGAGGAATTT | Phospho-N-acetylmuramoul-pentapetide | B488_07140 |
| CLIBASIA_05060 | -165 | ATAAAAAACGATTTTTAA | Hypothetical Protein | not found |
| CLIBASIA_05160 | -72 | TTATTTAATAAGTTTTAT | Hypothetical Protein | B488_07730 |
| CLIBASIA_05270 | -177 | ATAAACATTATTGATTAT | Uncharacterized HTH-type transcriptional regulator | not found |
| CLIBASIA_05275 | -145 | TTATAATTAAAGGAATAA | Hypothetical Protein | not found |
| CLIBASIA_05315 | -73 | TTAAGATTTGCGTTTTAA | Hypothetical Protein | not found |
| CLIBASIA_05365 | -62 | ATATACTAACAATATTAT | Enoyl-[acyl-carrier-protein] reductase | B488_02950 |
| CLIBASIA_05570 | -164 | ATAAACATTATGTTTTAT | Hypothetical Protein | not found |

^1^ Position is relative to the translational start point.

Table S3. Annotation of the selected genes for DNA binding assays from *L. asiaticus* and their homologs in *L. crescens*.

| ***L. asiaticus* locus_tag** | ***L. crescens* locus_tag** | **Blast2Go Annotation** |
| --- | --- | --- |
| CLIBASIA_01175 | B488_10900 | L,D-transpeptidase |
| CLIBASIA_01180 | B488_10910 | MarR family transcriptional regulator |
| CLIBASIA_00880 | B488_03970 | Metallophosphatase |
| CLIBASIA_01090 | B488_03160 | Alanine racemase |
| CLIBASIA_03450 | B488_00110 | DNA translocase |
| CLIBASIA_04090 | B488_10000 | dTDP-4-dehydrorhamnose 3,5-epimerase |
| CLIBASIA_04020 | B488_05250 | Glutamate racemase |
| CLIBASIA_02120 | B488_07960 | Zinc ABC transporter, periplasmic-binding protein ZnuA |
| CLIBASIA_02905 | B488_01790 | LuxR-family transcriptional regulator |
| CLIBASIA_03520 | B488_13750 | Septum formation protein Maf |
| CLIBASIA_04655 | B488_06440 | Kinesin-like protein |
| CLIBASIA_02120 | B488_10140 | Manganese ABC transporter, periplasmic-binding protein SitA |
| CLIBASIA_01505 | B488_01730 | Ferredoxin |
| CLIBASIA_03135 | B488_12950 | LotP |
| CLIBASIA_01670 | not found | Hypothetical protein |
| CLIBASIA_02420 | B488_11490 | ABC transporter permease |

Table S4. Oligonucleotides used in this study

| **Primer** | **Oligonucleotide sequence** | **Target** |
| --- | --- | --- |
| **EMSAs** |  |  |
| CLIB_01085_Fw | cgggattggtattgaaggatt | *L. asiaticus* |
| CLIB_01085_Rv^a^ | caatgttattgggagattcacg | *L. asiaticus* |
| CLIB_00880_Fw | ggctcacgatatcatggaaaa | *L. asiaticus* |
| CLIB_00880_Rv^a^ | ttttcaagtgttttgtttttgaca | *L. asiaticus* |
| CLIB_02905_Fw | aagagtaacatgactgttggtgtagtg | *L. asiaticus* |
| CLIB_02905_Rv^a^ | aaaacaatattcacctgctgtatttc | *L. asiaticus* |
| CLIB_03450_Fw | cggcaatagccatcacttct | *L. asiaticus* |
| CLIB_03450_Rv^a^ | tgttgccatttttattccttcc | *L. asiaticus* |
| CLIB_03520_Fw | tggaaaagataaatcaaacacttacc | *L. asiaticus* |
| CLIB_03520_Rv^a^ | tgcgtgataaactagaagatgc | *L. asiaticus* |
| CLIB_04090_Fw | atgcgatcaataaagcaacttat | *L. asiaticus* |
| CLIB_04090_Rv^a^ | aagtctgggaaaaccatcctc | *L. asiaticus* |
| CLIB_04655_Fw | acaaagtcatagaatacgctggt | *L. asiaticus* |
| CLIB_04655_Rv^a^ | tcaaactctttgagcgaatctc | *L. asiaticus* |
| CLIB_02120_Fw | gtttacaaataatctcaaattacac | *L. asiaticus* |
| CLIB_02120_Rv^a^ | atgccgacatgggaatata | *L. asiaticus* |
| CLIB_01670_Fw | gcacgatttacaagttcattcc | *L. asiaticus* |
| CLIB_01670_Rv^a^ | ttattgggagggaaatatttgtt | *L. asiaticus* |
| CLIB_04015_Fw | gggtttttctctttcccgaat | *L. asiaticus* |
| CLIB_04015_Rv^a^ | aattgaggggcatatggtgt | *L. asiaticus* |
| CLIB_01505_Fw | cccctcaatttcatcctcct | *L. asiaticus* |
| CLIB_01505_Rv^a^ | gcaattttcagtgacgacgta | *L. asiaticus* |
| CLIB_03135_Fw | gcaaggttgcgtcgtatctt | *L. asiaticus* |
| CLIB_03135_Rv^a^ | cggtaagtcttcacgatttttg | *L. asiaticus* |
| CLIB_02420_Fw | cgagaaataaacggtggtaaga | *L. asiaticus* |
| CLIB_02420_Rv^a^ | ccatctctttgggtgtaggaa | *L. asiaticus* |
|  |  | ‘ |
| **DNAse I footprinting** |  |  |
| CLIB_01670_Fw_FAM | gcacgatttacaagttcattcc | *L. asiaticus* |
| CLIB_01670_Rv | ttattgggagggaaatatttgtt | *L. asiaticus* |
| CLIB_04015_Fw_FAM | gggtttttctctttcccgaat | *L. asiaticus* |
| CLIB_04015_Rv | aattgaggggcatatggtgt | *L. asiaticus* |
|  |  |  |
| **qRT-PCR** |  |  |
| B488_10900_Fw | ggtggccacaggtggtaata | *L. crescens* |
| B488_10900_Rv | taacccatgtcgtgcttgaa | *L. crescens* |
| B488_10910_Fw | aacgtcaaggccgtagtgat | *L. crescens* |
| B488_10910_Rv | cgagcgctgatgattaacaa | *L. crescens* |
| B488_16S_Fw | cagaaccttaccagcccttg | *L. crescens* |
| B488_16S_Rv | attagctccgcctcacgact | *L. crescens* |
| B488_05250_Fw | ctgccgagcgaactttatct | *L. crescens* |
| B488_05250_Rv | ataggcacgcctgattgttc | *L. crescens* |
| B488_03840_Fw | gtaaagggcgctctggtatg | *L. crescens* |
| B488_03840_Rv | cggttcacatgcacaaaatc | *L. crescens* |
| B488_03160_Fw | cgtcccggaattgctattta | *L. crescens* |
| B488_03160_Rv | tttcccgccatagcttacag | *L. crescens* |
| B488_03970_Fw | gcgagagctcatccaacaat | *L. crescens* |
| B488_03970_Rv | tagctcgcgtagccttcttc | *L. crescens* |
| B488_01790_Fw | cagattgttggcaaacatgg | *L. crescens* |
| B488_01790_Rv | acttgccccaacgtactcag | *L. crescens* |
| B488_00110_Fw | aaatggatttggtgggatca | *L. crescens* |
| B488_00110_Rv | aagcaaaagctcctgttgga | *L. crescens* |
| B488_13750_Fw | catgactgacgctgaaaatca | *L. crescens* |
| B488_13750_Rv | cgcatttttaattttgcttctg | *L. crescens* |
| B488_10000_Fw | tgcacaggcaaaacttgttc | *L. crescens* |
| B488_10000_Rv | tctattccgacccaacaacc | *L. crescens* |
| B488_06440_Fw | cttctgaacgcgttgtttca | *L. crescens* |
| B488_06440_Rv | tgtttccagctcacttgcac | *L. crescens* |
| B488_01730_Fw | ttgtttttatgaaggcgaaaa | *L. crescens* |
| B488_01730_Rv | ttgttctcaaccccatccat | *L. crescens* |
| B488_10140_Fw | ccaacaagttcgcaaagtca | *L. crescens* |
| B488_10140_Rv | tattttgctccggtttccag | *L. crescens* |
| B488_07960_Fw | gaaacgcatcgccattatct | *L. crescens* |
| B488_07960_Rv | caattttgccatgtttgctg | *L. crescens* |
| B488_12950_Fw | cgttgtttttatattgctccgtttg | *L. crescens* |
| B488_12950_Rv | cacggtctacaccgctatcttct | *L. crescens* |
| *ldtR*_Fw | atttcggacacggatagtgc | *L. asiaticus* |
| *ldtR*_Rv | tgtaatcgctcaaccaaacg | *L. asiaticus* |
| CLIBASIA_02905_Fw | ctccacctgctggtatggat | *L. asiaticus* |
| CLIBASIA_02905_Rv | gcagcgagcaaacctatttc | *L. asiaticus* |
| CLIBASIA_04655_Fw | gcacgatgcttctcaatcaa | *L. asiaticus* |
| CLIBASIA_04655_Rv | ctcttccgtcatcagcacaa | *L. asiaticus* |
| CLIBASIA_01505_Fw | gcatacagattgcgtggaagt | *L. asiaticus* |
| CLIBASIA_01505_Rv | ccccacaatctatgcactcat | *L. asiaticus* |
| CLIBASIA_01670_Fw | tcaatttagtacgtggaggcag | *L. asiaticus* |
| CLIBASIA_01670_Rv | tcccttcttttgtcagagctg | *L. asiaticus* |
| CLIBASIA_04020_Fw | atccatggcttttcttggtg | *L. asiaticus* |
| CLIBASIA_04020_Rv | gtcgcaggcgttgataaaat | *L. asiaticus* |
| CLIBASIA_00325_Fw | tatcccaatgtgctggtcaa | *L. asiaticus* |
| CLIBASIA_00325_Rv | gacccgttgcataagcattt | *L. asiaticus* |
| CLIBASIA_r05781(16S)_Fw | tcgagcgcgtatgcgaatacg | *L. asiaticus* |
| CLIBASIA_r05781(16S)_Rv | gcgttatcccgtagaaaaaggtag | *L. asiaticus* |

^a^ Biotin labeled

Figure S1. Correlation between the fold changes of the differentially expressed genes identified in the RNAseq experiments. The linear regression of the fold change values obtained in the benzbromarone and phloretin treatments was calculated in MicroCal Origin 9.0.


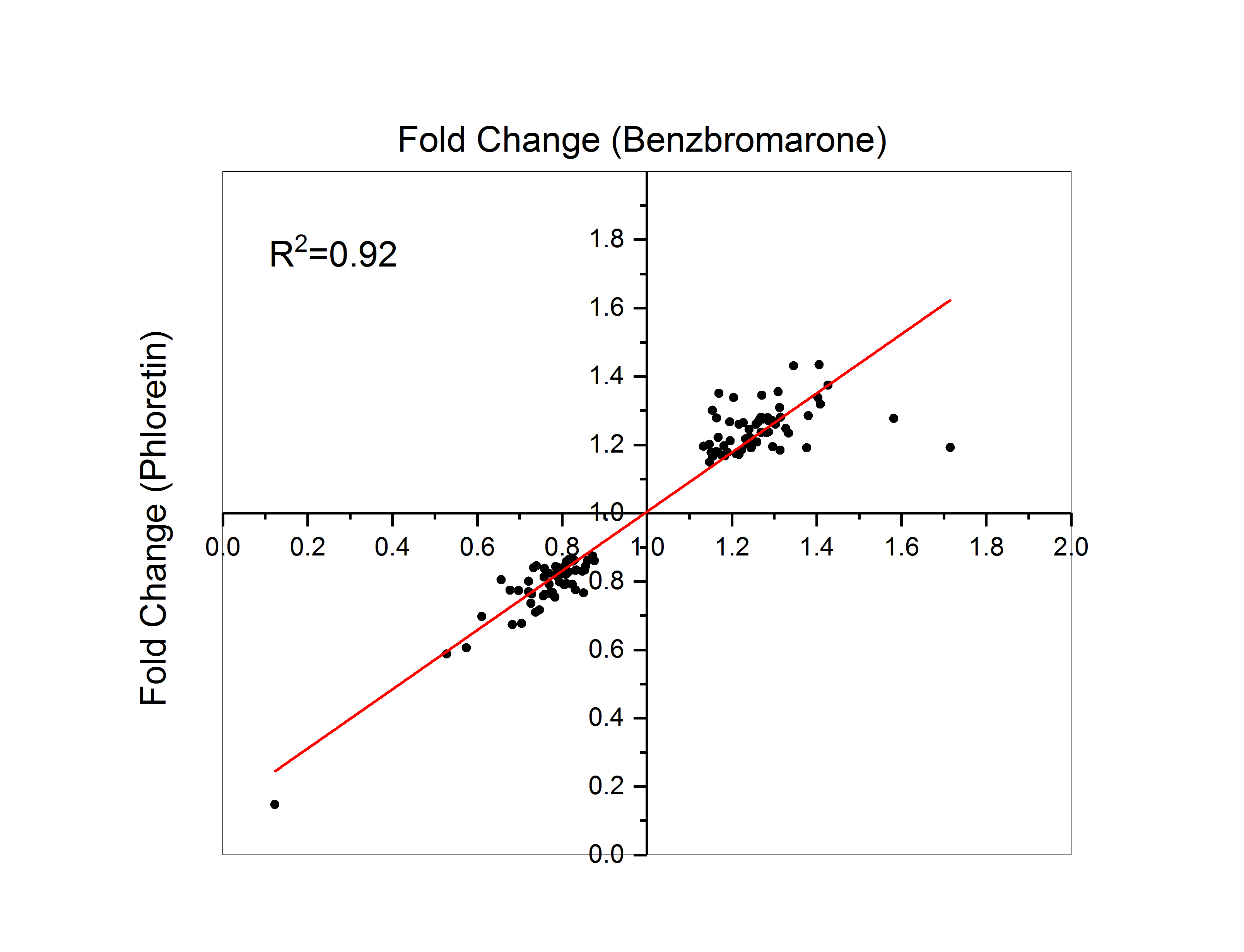


Figure S2. LdtR ligands disrupt LdtR binding to *P_CLIBASIA_02120_* and *P_CLIBASIA_03520_.* EMSAs were conducted in the absence or presence of either benzbromarone or phloretin, as indicated on top of each panel. Protein was not added to the first lane.


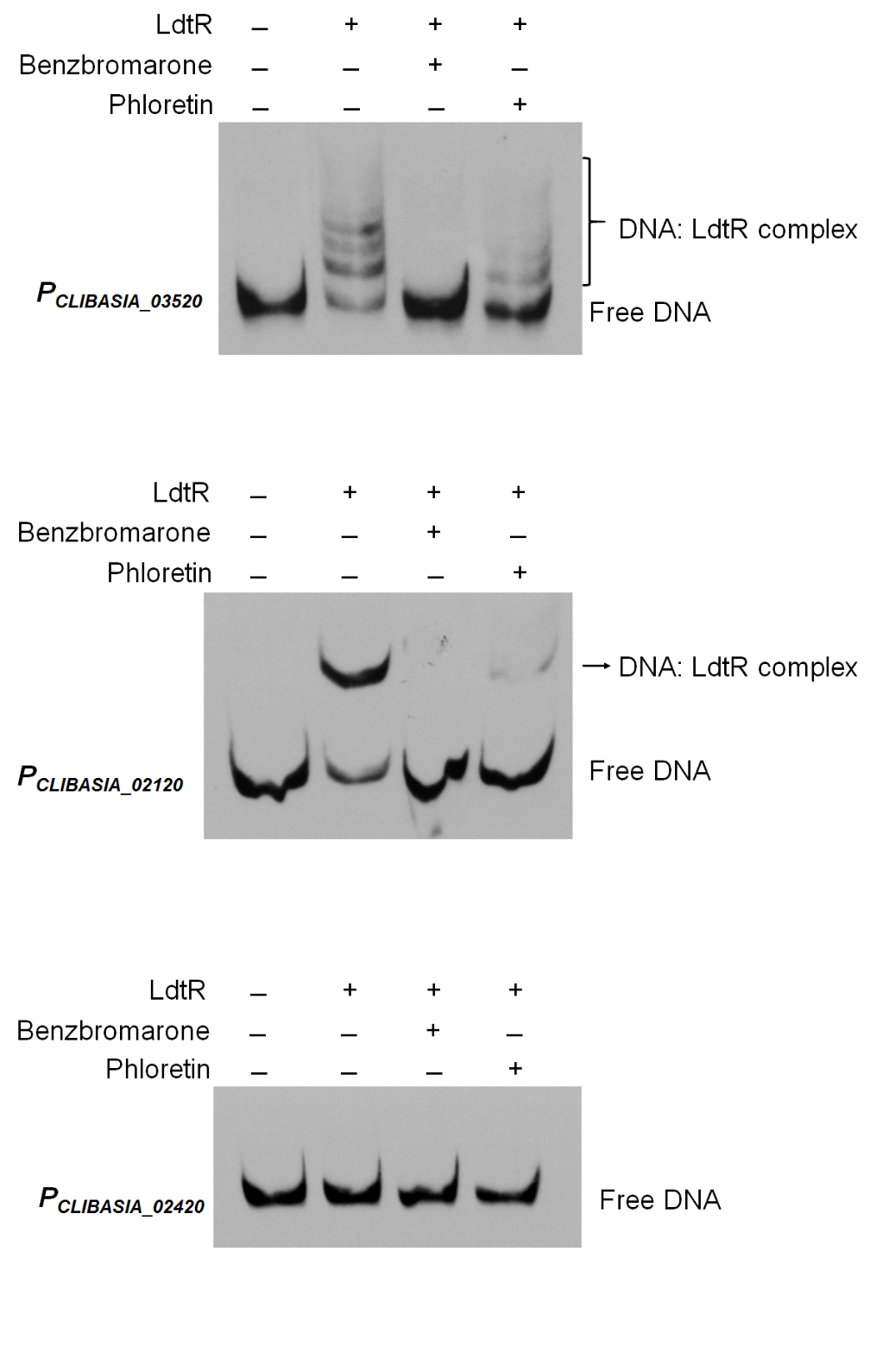

Supplement: Supplementary file 1 — Table S1. List of genes differentially expressed in L. crescens treated with 20 µM phloretin. Table S2. The location of the putative LdtR binding sites in L. asiaticus genome not identified in the RNA‐seq experiments. Table S3. Annotation of the selected genes for DNA binding assays from L. asiaticus and their homologs in L. crescens. Table S4. Oligonucleotides used in this study. Figure S1. Correlation between the fold changes of the differentially expressed genes identified in the RNAseq experiments. The linear regression of the fold change values obtained in the benzbromarone and phloretin treatments was calculated in MicroCal Origin 9.0. Figure S2. LdtR ligands disrupt LdtR binding to P CLIBASIA_02120 and P CLIBASIA_03520. EMSAs were conducted in the absence or presence of either benzbromarone or phloretin, as indicated on top of each panel. Protein was not added to the first lane. [file MBT2-10-896-s001.docx]
